# Driving-Context Classification from Wearable Physiological and Motion Signals During Real-World Driving

**DOI:** 10.3390/s26196202

**Published:** 2026-09-30

**Authors:** Poh Ping Em, Tai Shie Teoh

**Affiliations:** Faculty of Engineering and Technology, Multimedia University, Jalan Ayer Keroh Lama, Melaka 75450, Malaysia; 1151102396@student.mmu.edu.my

**Keywords:** wearable sensors, electrodermal activity, heart rate variability, driver monitoring, driving context classification, machine learning, leave-one-driver-out cross-validation, mixed effects models, pseudo-replication

## Abstract

Wearable physiological sensing is widely used in driving research to infer drowsiness and stress, but whether driving context itself is reflected in wearable signals, independent of any drowsiness label, is rarely examined directly; any such signal reflects the driver’s physiological response to context, not the road environment itself. We analyzed 1536 non-overlapping 60-s windows from 18 driving sessions completed by 11 drivers wearing an Empatica EmbracePlus across three fixed real-world routes (Rural, Highway, Urban; round-trip distances 22.4–47.1 km). Because these windows are correlated pseudo-replicates of the 18-session experimental unit rather than independent observations, we report both the window-level mixed-effects comparison a naive analysis would present as primary (27 of 32 features significant after false-discovery-rate correction) and a corrected session-level analysis with driver-clustered standard errors, covariate adjustment for trip duration, sleep, pre-drive sleepiness, and route order. Using a single omnibus test per feature, only 2 of 32 features (skin conductance response count and accelerometer movement count) remain significant once the statistical unit matches the experimental unit and pairwise multiplicity is properly controlled. A random forest evaluated with leave-one-driver-out cross-validation achieved only 34.9% accuracy (macro-F1 = 0.33; 95% CI [20.0%, 49.9%]) against a 27.0–32.7% baseline, versus 90.6% accuracy (95% CI [89.1%, 92.0%]) under a naive ungrouped cross-validation that leaks driver identity across folds; the 55.6-point gap is clearly distinguishable from zero (95% CI [42.0, 69.2]). A four-way modality ablation (physiology, accelerometry, temperature, all combined) found no dominant sensor channel and the combined-channel model did not outperform accelerometry; these results are consistent with a diffuse cross-modal signature rather than a physiology-specific one. Driving context is statistically distinguishable from wrist-worn wearable signals once analyzed at the correct statistical unit, but the signal is diffuse across sensor channels, and person-independent classification remains weak at this sample size; naive cross-validation and uncorrected pairwise-multiplicity designs common in this literature can substantially overstate both classification and statistical testing results.

## 1. Introduction

Wrist-worn wearables such as the Empatica E4 and EmbracePlus are increasingly used in driving research to capture electrodermal, cardiovascular, and motion signals during real-world and simulated drives [1,2,3]. The dominant application of this signal stream is drowsiness and fatigue detection: several reviews and empirical studies show that wearable-derived heart rate, HRV, and EDA features carry information about a driver’s state of alertness [4,5,6,7]. A separate line of work uses the driving task itself, and specifically its varying cognitive and manual demand, to probe how HRV and EDA shift under load, typically comparing single- versus dual-task driving or specific maneuvers such as intersection turns [8,9,10,11].

A related but distinct question, and the one this paper addresses, is whether driving context itself, independent of any drowsiness or workload label, leaves a detectable signature in wearable physiological and motion signals. This question matters for two reasons. First, driving context is a plausible confound for drowsiness and stress models trained on naturalistic or on-road data: if EDA or HRV shift systematically between a monotonous highway and a demanding urban corridor, a drowsiness classifier that ignores driving context risks attributing environmental variance to driver state. Second, motion-based road-surface and terrain classification is an established problem in intelligent transportation systems, but it is almost always solved with sensors rigidly coupled to the vehicle chassis or a smartphone mounted in the cabin [12,13,14,15,16] rather than a wrist-worn device where the accelerometer signal is filtered through the arm and body. Whether a device already being worn for physiological monitoring can also carry a usable driving context signal without any additional instrumentation is an open and practically relevant question.

For terminological clarification, this paper uses Rural, Highway, and Urban as route or driving environment categories, not road surface conditions in the sense used in the terrain and road surface classification literature reviewed in Section 2 (pavement roughness, potholes, gravel, and similar physical surface properties). Maps and camera-based systems can already identify objective road surface types directly, typically more reliably than a wrist-worn sensor could. The practically useful application of a positive result here is not competing with that literature on road surface sensing but instead demonstrating that driving context and everything correlated with it (speed, traffic, trip duration, route geometry) can contaminate wearable-based driver state models that are not designed to account for it. We use the term “driving context” throughout to keep this distinction clear, and reserve “road condition” for the literal dataset field name inherited from the data collection protocol where a code-style reference to that field is needed.

A second clarification concerns what a wrist-worn physiological sensor can and cannot measure. It cannot detect an objective driving context category directly; it measures the driver’s physiological response, and any association between that response and driving context is necessarily mediated by constructs such as cognitive workload, perceived demand, arousal, or situational awareness [17,18]. We did not collect independent workload, drowsiness, or situational awareness labels in this study, so the absence of a directly measured mediator does not establish that the physiological differences reported here are independent of these states. We frame our findings throughout as associations between driving context and wearable signal, mediated by the driver’s physiological and attentional response, rather than as direct sensing of the road, and return to this distinction when interpreting the statistical and classification results (Section 4 and Section 5).

We use a dataset of 60-s windows collected from 11 drivers wearing an Empatica EmbracePlus across Rural, North–South Highway, and Urban routes, then ask whether driving context be classified, or at least meaningfully distinguished, from wearable physiological and motion signals alone. We deliberately treat this as a classification and statistical separability question rather than a drowsiness-adjacent one, and we give particular attention to methodological issues that are easy to overlook in small repeated-measures wearable datasets: whether apparent classification accuracy reflects a context-general signal or simply the classifier learning to recognize individual drivers, and whether apparent statistical significance reflects a real effect or a mismatch between the 1536-window statistical unit and the 18-session experimental unit. The paper’s contributions are: (1) a population-level statistical comparison of 32 wearable-derived features across three driving contexts, reported both at the window level with a driver-only random intercept and at the corrected session level, with driver-clustered standard errors, covariate adjustment, and a single omnibus test per feature in place of an uncorrected pairwise-minimum procedure; (2) a driver-grouped (leave-one-driver-out) classification evaluation, contrasted directly against a naive ungrouped cross-validation to quantify how much pseudo-replication inflates apparent accuracy; (3) permutation and SHAP feature importance under the driver-grouped scheme; (4) a within-subject sensitivity analysis restricted to drivers who completed more than one route; (5) a four-way modality ablation (physiology, accelerometry, temperature, all combined) testing whether classification performance is specific to any one sensor channel; and (6) a causal prior-session-only baseline correction analysis for the seven multi-route drivers, replacing an earlier transductive version that was calibrated on data including the held-out test windows.

## 2. Related Work

### 2.1. Physiological Correlates of Driving Demand and Road Context

Heart-rate variability has long been used as an index of mental workload in driving and other vehicle control tasks. Comparisons of single- versus dual-task driving [8], visual workload estimation combining HRV with gaze [9], and workload changes during specific maneuvers such as intersection turns [10] and secondary in-vehicle tasks [11] consistently report HRV shifts with driving demand. HRV has also been examined for driver sleepiness classification directly from real road recordings [19], and heart rate together with skin conductance has been shown to reveal distinct aspects of cognitive load within a single driving scenario [20]. More general physiology work confirms that mental workload measurably alters HRV’s nonlinear dynamics outside the driving context [21], supporting HRV as a workload-sensitive signal, though not on its own establishing whether workload differences track road type versus task demand imposed by the experimenter. A recent systematic review of in-vehicle physiological indices and sensor technology for driver mental workload monitoring [17] surveys this broader literature and likewise notes that road and driving-context factors are rarely disentangled from workload per se, which is the gap this paper addresses.

### 2.2. Electrodermal Activity in Driving Contexts

EDA is a standard index of sympathetic arousal [22] and its use as a stress indicator during driving dates to early instrumented-vehicle studies [23]. More recent work has measured EDA in driving simulators [24], linked EDA and vehicle control data to driver emotion during simulated driving [25], and examined EDA responses to automated driving interface uncertainty [26]. A simulated driving emotion recognition framework built directly on EDA features [27] and general-purpose EDA feature extraction and stress detection pipelines [28,29] establish the feature families (tonic/phasic decomposition, SCR count and amplitude) used in the present study. Random forest classification from EDA has separately been validated for emotion recognition outside the driving domain [30], motivating the same classifier family here. More recent work continues to tie EDA directly to driving context: EDA has been measured during distracted driving in a simulator [31], used with random forest recursive feature elimination to distinguish driver behavior events [32], and linked via association rule mining to speed variation at road intersections specifically [33], the last of these being closely related to the road context question addressed here, though it examines within-route speed variation rather than road type discrimination. A separate methodological finding, that EDA collected from a wrist-worn device can show systematic left–right (lateralization) differences [34], is a relevant caveat for any single-wrist wearable EDA measurement, including the present study.

### 2.3. Motion and Accelerometer-Based Road and Terrain Classification

Estimating road surface condition or terrain type from accelerometer signals is a mature sub-area of intelligent transportation systems research. Smartphone-based approaches have used accelerometer and gyroscope data to estimate road roughness [15], detect road surface anomalies in a federated learning setting [35], and classify surface condition with a range of machine learning pipelines [16,36,37,38,39], including kernel-based sequence methods [40]. Vehicle- or platform-mounted inertial sensors have been used for terrain and road surface type classification more broadly [12,13,14,41,42,43] and for online road roughness classification from unsprung-mass acceleration [44]. This literature consistently reports strong classification performance, but relies on sensors rigidly coupled to the vehicle or held in a fixed position, which removes the additional damping and voluntary movement noise that a wrist-worn sensor introduces. More recent accelerometer-based road surface work continues in this vein, including ultra-low-power on-device classification [45], convolutional models combining accelerometer and speed data [46], smartphone-based anomaly detection under concept drift [47], multimodal Bayesian filtering [48], dual-filter IMU sensor fusion [49], and comparisons of vibration signal data representations [50]. Like the earlier work above, all of these use vehicle- or device-fixed accelerometers rather than a wrist-worn sensor.

### 2.4. Wearable Sensors in Transportation Research and Naturalistic Driving

Wearable and body-worn sensing has been integrated with driving simulators [1,3] and tested in initial real-road studies [2], including for wearable-based drowsy driving detection [51]. Naturalistic driving studies more broadly, including the SHRP2 [52] and 100-Car [53] datasets and related naturalistic observation methodology [54,55], established the value of unscripted real-world driving data, though these large corpora do not include wrist-worn physiological sensing. Wearable monitoring of psychological and physiological state has also been studied in other naturalistic (non-driving) settings [56]. Reviews of intelligent driver monitoring systems built on physiological sensors [57] and more recent work combining wearable sensing with explainable AI for cognitive load monitoring [58] frame the broader context this study sits within. The closest direct predecessor to the present study’s research question is a single-subject 21-day longitudinal case study that used EDA from an Empatica E4 to classify road type (highway vs. city), achieving approximately 82.9% accuracy for the road type model [59]. Because that design used one driver, it carries no between-driver confound and is not directly comparable to a person-independent evaluation; however, it establishes that road type is decodable from EDA within a single well-sampled individual, a result we return to when interpreting our own within-subject sensitivity analysis in Section 5. More recent work on wearables in transportation extends this line of research to electric vehicle driving confidence monitoring [60], IoT-connected truck driver stress monitoring [61], integrated facial-plus-physiological driver behavior monitoring [62], and a multimodal driver monitoring benchmark dataset for assisted driving research [63]. A recent review of driver behavior under naturalistic driving conditions [64] reinforces the value of unscripted real-world data, as noted above. A wearable EDA-and-HRV workload study in a simulated railway signaling task [65] shows the same wearable workload paradigm generalized to a non-driving transportation domain. A closely related but conceptually distinct body of work uses wearable physiological and motion sensors specifically to detect drowsiness [4,5,6,7,66], including a recent domain generalization approach to physiological drowsiness estimation in conditional automated vehicles [67]. Like the present study, it targets generalization across drivers rather than within-driver accuracy; however, we deliberately separate our driving context question from their framing, since otherwise a driving context signal could, if present, be mistaken for or entangled with a drowsiness signal in prior work. Most directly related to the present study is work that combines drowsiness detection with real-time road-condition detection from the same sensor set, heart rate, accelerometer, and gyroscope [68]. Unlike that work, however, we isolate driving context as the sole target and evaluate it under a driver-grouped design independently of any drowsiness label.

### 2.5. Validation of Empatica Wearables for Physiological Measurement

Because signal quality from a consumer wrist wearable is central to interpreting our results, we draw on the validation literature for the Empatica device family. The Empatica E4 wristband, which shares sensor hardware with the newer EmbracePlus, has been validated against research-grade equipment for electrodermal activity [69,70,71], cardiac and electrodermal measures jointly [72], and EDA/HRV in interactive settings [73]. E4-based stress detection pipelines and public datasets [74,75] as well as broader open-source wearable validation work [76] further support the feature families used here. Most directly relevant, a recent multi-device reliability study evaluated the EmbracePlus alongside the Shimmer3 GSR+, Empatica E4, and Pixel Watch 2 across cognitive, affective, and physical activity tasks [77], providing current evidence for the signal quality of the device used in this study.

## 3. Materials and Methods

**Figure 1:**
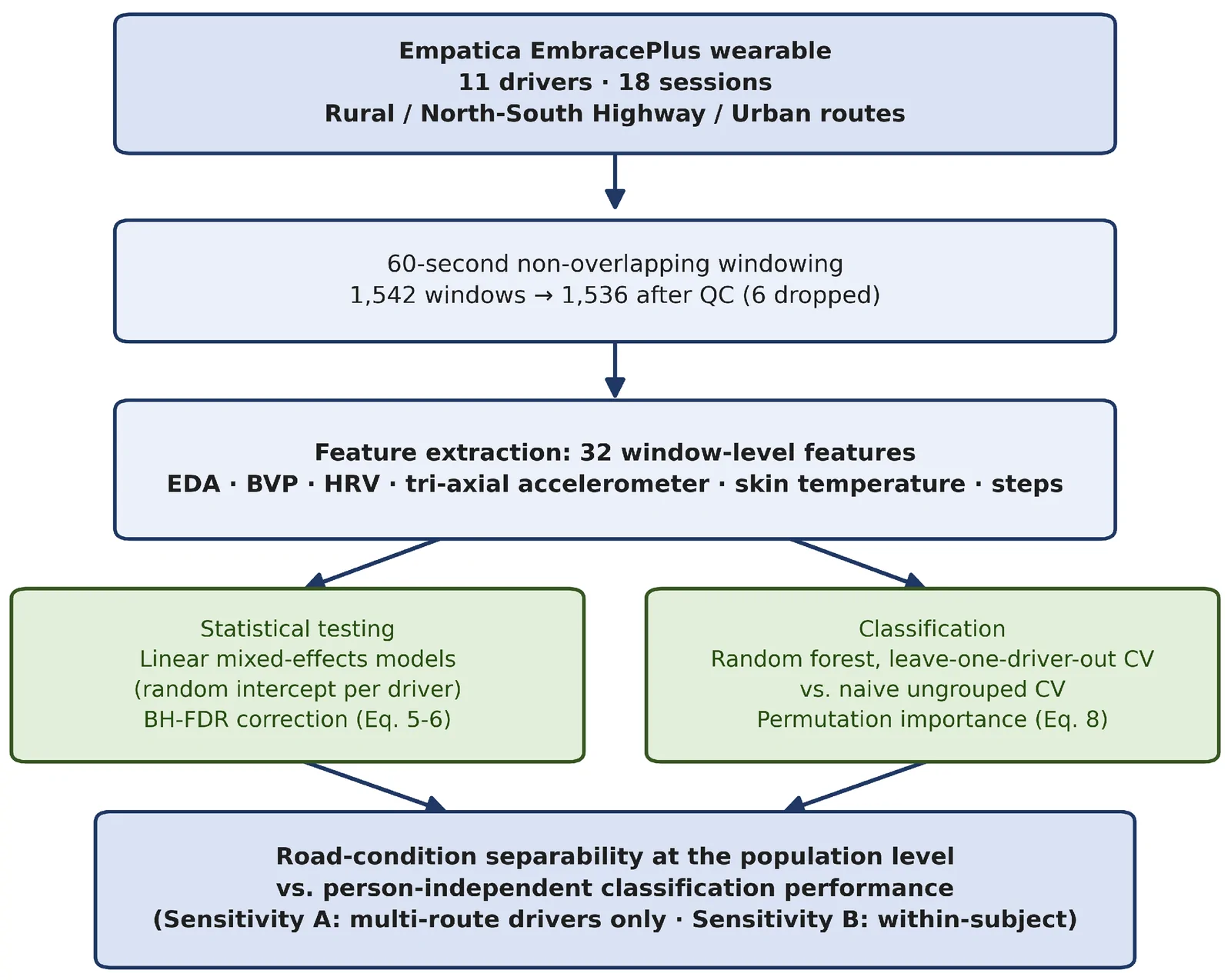
Overview of the data processing and analysis pipeline, from raw EmbracePlus sensor acquisition through window-level feature extraction to the parallel statistical testing and driver-grouped classification analyses.

Figure 1 summarizes the overall analysis pipeline described in this section, from raw sensor acquisition through windowing and feature extraction to the two parallel statistical and classification analyses and their associated sensitivity checks.

### 3.1. Dataset

The dataset comprises 1542 non-overlapping 60-s windows drawn from 18 driving sessions completed by 11 drivers (9 male, 2 female; 9 in the 18–35 age group, 2 aged over 56; self-reported driving experience of under 5, 5–10, or over 10 years) wearing an Empatica EmbracePlus wearable (Empatica Inc., Boston, MA, USA). All sessions were conducted in the same vehicle (a 2014 Perodua Axia; Perodua, Rawang, Selangor, Malaysia). The EmbracePlus was worn on whichever wrist each participant normally wears a watch on, predominantly the left wrist but self-selected rather than standardized across participants; this detail is relevant to the electrodermal activity wrist lateralization caveat discussed in Section 2 in relation to [34]. Each session was labeled with one of three road_condition types: Rural, North–South Highway, or Urban. Seven of the eleven drivers completed more than one route type (five drove both Highway and Urban, one drove both Rural and Highway, and one drove both Rural and Urban), while the remaining four drivers completed only the Rural route. Six windows (0.4% of the dataset) had at least one missing sensor feature and were dropped, leaving 1536 windows for analysis: 553 Highway, 541 Urban, and 442 Rural. Table 1 shows the window count by driver and route.

**Figure 2:**
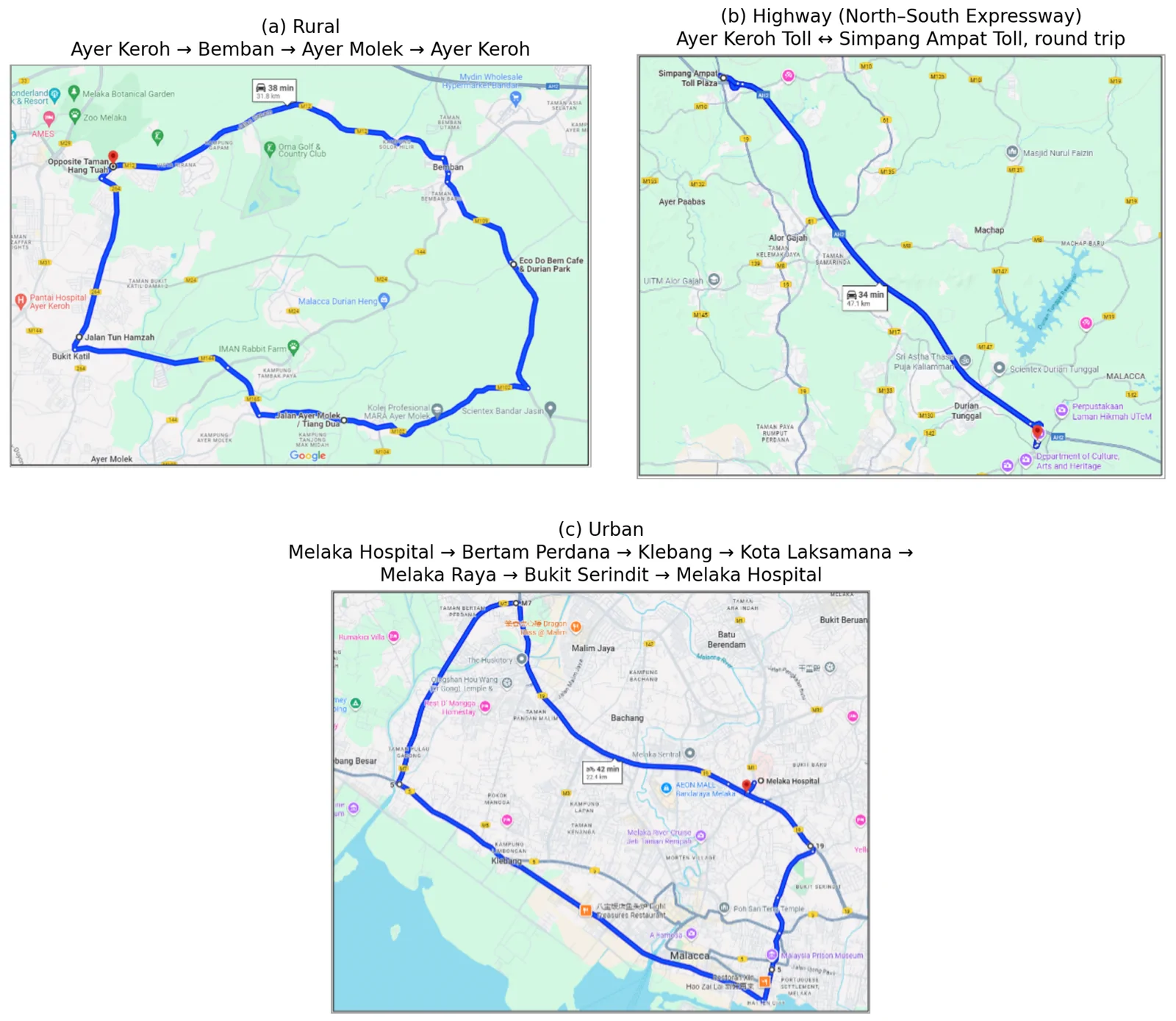
Google Maps driving directions for the three fixed routes, matching the route planning documentation used to brief participants; the on-screen distance and duration match the planned values reported in the text. Map data © Google.

Each route was a fixed pre-planned round trip confirmed against the route planning documentation used to brief the participants. Highway (North–South Expressway, Ayer Keroh Toll–Simpang Ampat Toll–Ayer Keroh Toll, round trip) covered 47.1 km in a nominal 34 min (implied average speed 83.1 km/h); Rural (Ayer Keroh–Bemban–Ayer Molek–Ayer Keroh, loop) covered 31.8 km in 38 min (50.2 km/h); Urban (Melaka Hospital–Bertam Perdana–Klebang–Kota Laksamana–Melaka Raya–Bukit Serindit–Melaka Hospital, loop) covered 22.4 km in 42 min (32.0 km/h). All sessions were conducted in the afternoon (1–4 pm). These planned distances, durations, and implied speeds are reported here as descriptive context for interpreting the physiological differences in Section 5. Because each takes exactly one constant value per driving-context category, all three are perfectly collinear with the driving context label itself and cannot serve as adjustment covariates in the statistical model below (Section 3); they are included only as narrative interpretation. Figure 2 shows each route as planned in Google Maps (web version; Google LLC, Mountain View, CA, USA).

### 3.2. Feature Extraction Summary

We used 32 window-level features extracted from four sensor streams as model inputs: electrodermal activity (EDA) mean, standard deviation, minimum, maximum, and slope of the raw signal; mean and slope of the tonic (slow-varying) component; standard deviation of the phasic (fast-varying) component; and skin conductance response (SCR) count and mean amplitude. These follow the standard decomposition of the EDA signal into a slow-varying tonic (skin conductance level) component and a fast-varying phasic (skin conductance response) component:

(1) $$EDA\left(t\right)=EDA_{\text{tonic}}\left(t\right)+EDA_{\text{phasic}}\left(t\right),$$

where both tonic and phasic components, along with the raw signal, contribute separate summary features (Equation (1)).

Blood volume pulse (BVP): mean, standard deviation, minimum, and maximum. Heart rate variability, derived from systolic peaks detected in the BVP signal: peak count, mean heart rate (bpm), mean inter-beat interval (IBI), SDNN, RMSSD, and pNN50, computed from the sequence of *N* inter-beat intervals in each 60-s window as

(2) $$SDNN=\sqrt{\frac{1}{N-1}\sum_{i=1}^{N}{\left(IBI_{i}-IBI_{\text{avg}}\right)}^{2}},$$

(3) $$RMSSD=\sqrt{\frac{1}{N-1}\sum_{i=1}^{N-1}{\left(IBI_{i+1}-IBI_{i}\right)}^{2}},$$

(4) $$pNN50=\frac{NN50}{N-1}\times 100\%,$$

where $IBI_{\text{avg}}$ is the mean inter-beat interval for the window and NN50 is the count of successive IBI pairs differing by more than 50 ms (Equations (2)–(4)). Tri-axial accelerometry: mean and standard deviation of the acceleration magnitude vector, its maximum, the standard deviation on each individual axis, and a movement count feature. Skin temperature: mean, standard deviation, and slope. Two additional window-level features, namely, cumulative step count and tag/event count, were included as auxiliary motion and annotation covariates. All 32 features were used together as the input feature space for both the statistical and classification analyses; session metadata (participant ID, date, demographics) were retained for grouping and description but excluded from the feature set given to the classifier. Feature extraction from the raw EmbracePlus data stream followed the same general processing conventions (signal decomposition and windowed summary statistics per sensor channel) documented for open-source EmbracePlus processing software [78].

### 3.3. Signal Quality Criterion

No artifact rejection or peak detection validity check was applied in the original feature extraction pipeline. Because wrist motion during driving can plausibly degrade blood volume pulse (BVP) peak detection, and with it every heart rate variability (HRV) feature derived from the inter-beat interval sequence, we implemented a minimum valid beat count criterion before using these features in the statistical or classification analyses. The distribution of peak_count (systolic peaks detected per 60-s window) has a clear isolated gap: twelve windows (0.78% of the 1536-window dataset, spread across 12 of the 18 sessions, never more than one per session) have peak_count below 50, several with hr_mean_bpm or ibi_mean_ms values physiologically inconsistent with that few detected beats (e.g., one window with six detected peaks reports a mean heart rate of 99.4 bpm, which six beats in 60 s cannot support); the distribution resumes as a continuous run from 51 peaks upward. This pattern is consistent with PPG dropout or motion artifact rather than genuine bradycardia in an actively driving population. We treat peak_count < 50 as invalid for HRV-derived features specifically (hr_mean_bpm, ibi_mean_ms, sdnn_ms, rmssd_ms, pnn50_pct), excluding values from these twelve windows for those five features from all downstream means and models; peak_count itself and the raw BVP waveform statistics, which do not require valid peak detection to compute, are unaffected. Applying this criterion left the session-level statistical results (Section 4) unchanged and changed the primary leave-one-driver-out classification accuracy by less than half a percentage point (34.9% to 34.6%), so none of this paper’s main findings depend on it; we report the criterion and its negligible impact here as a methodological completeness check on the HRV-derived features specifically.

### 3.4. Analysis Approach

**Figure 3:**
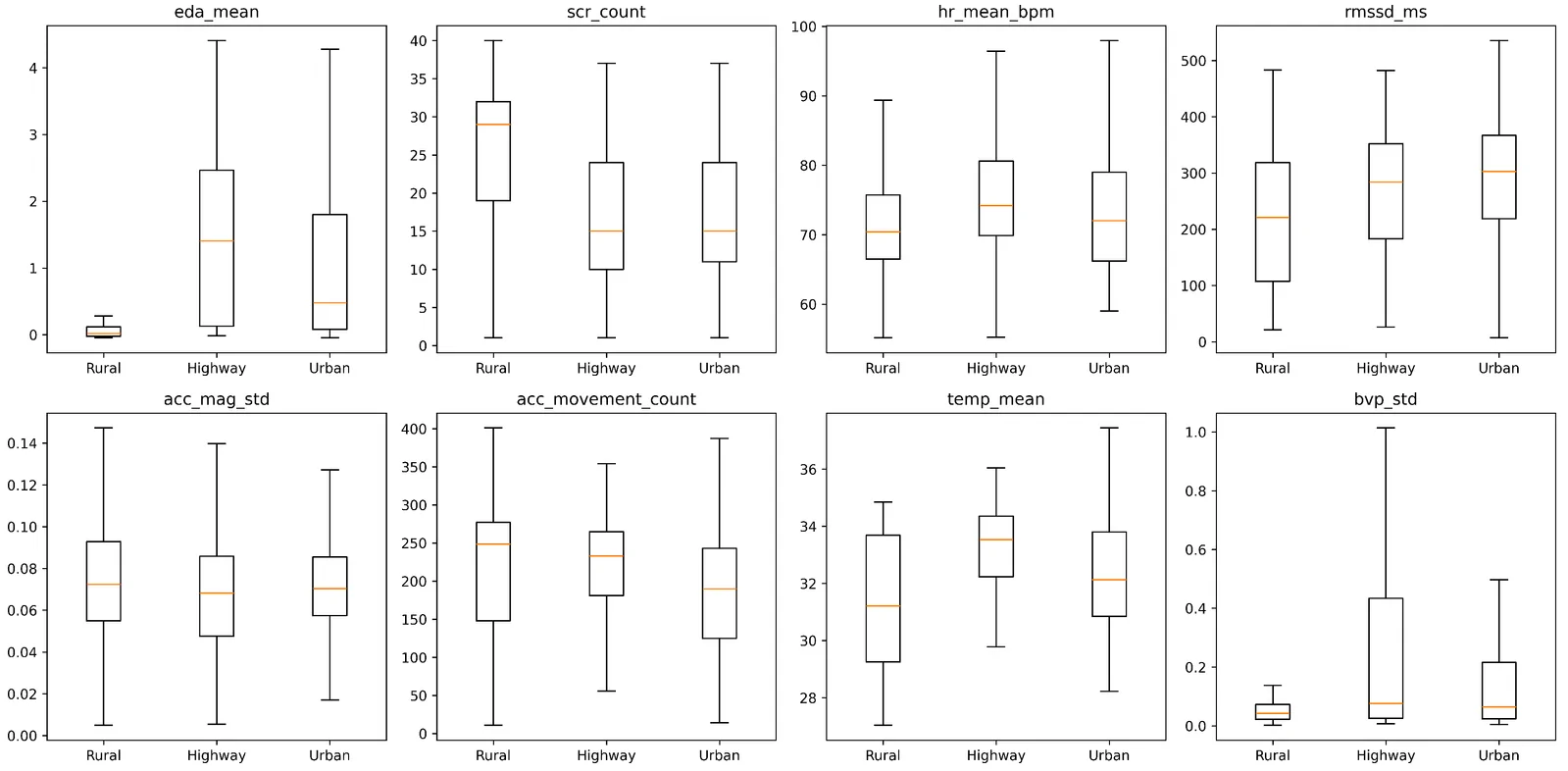
Distributions of eight representative features (one per sensor modality) by driving context, outliers omitted for readability.

*Descriptive comparison.* Mean and standard deviation for all 32 features were computed by driving context, and distributions for eight representative features spanning all four sensor modalities are shown as boxplots (Figure 3).

*Statistical testing (window level).* For each feature we fit a linear mixed-effects model (LMM) with road_condition as a three-level fixed effect and a random intercept for participant_id, which accounts for repeated measurements within driver but treats each of the 1536 60-s windows as a separate observation. Writing $y_{ijk}$ for the value of a given feature in window *k* of driver *i* on route *j*, the model specification is

(5) $$y_{ijk}=\beta_{0}+\beta_{1}\;I\left(\text{Highway}\right)+\beta_{2}\;I\left(\text{Urban}\right)+u_{i}+\varepsilon_{ijk},$$

(6) $$u_{i}∼N\left(0,\sigma_{u}^{2}\right),\;\varepsilon_{ijk}∼N\left(0,\sigma^{2}\right),$$

where I(Highway) and I(Urban) are indicator variables for route type (Rural as the reference level), $u_{i}$ is the driver-level random intercept, and $\varepsilon_{ijk}$ is the residual error (Equations (5) and (6)). Because a single likelihood-ratio test comparing the full model against a random intercept-only null model was numerically unstable for several features (boundary/near-zero random effect variance), we instead report pairwise Wald tests from the fitted model, obtained by fitting the model twice with different reference levels (Rural and Highway) to recover all three pairwise contrasts (Rural vs. Highway, Rural vs. Urban, Highway vs. Urban). Each feature’s overall significance is summarized by the minimum *p*-value across its three pairwise contrasts, and Benjamini–Hochberg false discovery rate (FDR) correction was applied across all 32 features. We report this window-level procedure because it is the analysis a study of this design would typically present as primary, and because contrasting it against the corrected procedure below (Section 4) quantifies the size of the two problems that procedure addresses.

*Statistical testing (session level, primary).* The window-level model above has two problems, both raised independently in review: first, road_condition is assigned at the session level, so the 1536 windows are correlated pseudo-replicates of only 18 true sessions, not 1536 independent observations; a driver-only random intercept does not correct for this, since it operates at the wrong level of the data hierarchy. Second, taking the minimum of three pairwise *p*-values per feature and then applying FDR correction across features does not control the pairwise multiplicity within a feature before the cross-feature correction is applied, thereby inflating the apparent number of significant features. We address both of these concerns by repeating the analysis at the session level. Each of the 32 features is aggregated to its 18 session-level means, and we fit

(7) $$y_{i}=\beta_{0}+\beta_{1}\;I{\left(\text{Highway}\right)}_{i}+\beta_{2}\;I{\left(\text{Urban}\right)}_{i}+\gamma^{⊤}c_{i}+\varepsilon_{i}$$

by ordinary least squares with driver-clustered robust standard errors (eleven driver clusters, four of which are singletons because four drivers contributed only one session), where $c_{i}$ is the vector of session-level covariates: trip duration (window count for that session), hours slept, pre-drive Karolinska Sleepiness Scale score, and within-driver chronological route order (Equation (7)). These four variables are the only previously unused ones that genuinely vary within road_condition category, meaning that they can function as adjustment covariates. The route-level Google Maps distance, duration, and implied speed reported in Section 3 (Dataset) each take exactly one constant value per road_condition category, and are consequently perfectly collinear with the driving context label itself; thus, we do not include them as covariates here. For each feature, we tested the omnibus null hypothesis that driving context has no effect at all via a single cluster-robust Wald *F*-test on both driving context coefficients jointly, and applied FDR correction across the resulting 32 omnibus *p*-values. For features that passed this FDR gate, we additionally report the three pairwise contrasts as Holm-corrected post hoc comparisons. These comparisons are descriptive only of which specific pairs differ. Because cluster-robust inference with only eleven clusters (four of them singleton) sits at the edge of reliable small-sample asymptotics, we cross-checked the omnibus-significant features with a 5000-draw label permutation test (road_condition shuffled across the 18 sessions, the cluster-robust omnibus *F*-statistic recomputed on each draw, empirical *p* = the proportion of permuted statistics at or above the observed value).

*Modality ablation.* To test the implications of the multicollinearity diagnostic in Section 4.3 for classification, i.e., whether performance is genuinely cross-modal or driven by a single sensor channel, we separately repeated the identical leave-one-driver-out random forest evaluation described below with the same hyperparameters and scoring on four feature subsets: physiology (twenty features, electrodermal activity plus BVP/heart rate variability), accelerometry (eight features, tri-axial accelerometer statistics plus step count), temperature (three features, skin temperature mean, standard deviation, and slope), and all modalities combined (31 features, tags_count excluded as constant).

*Classification.* A random forest classifier (300 trees, minimum leaf size 3, balanced-subsample class weighting) was trained to predict road_condition from the 32-feature vector. Because windows from the same driver are not independent, the primary evaluation used leave-one-driver-out cross-validation (eleven folds via scikit-learn’s LeaveOneGroupOut, grouped by participant_id): in each fold, all windows from one driver were held out for testing and the model was trained on the remaining ten drivers. We report overall accuracy, macro-averaged F1 across the C=3 driving-context classes:

(8) $$F1_{\text{macro}}=\frac{1}{C}\sum_{c=1}^{C}F1_{c},$$

and the confusion matrix pooled across folds (Equation (8)) alongside majority-class and stratified-random dummy classifier baselines evaluated under the identical leave-one-driver-out scheme. Feature importance was computed as permutation importance on each fold’s held-out data and averaged across folds: for feature *j*, scoring function *s*, and *K* random permutations,

(9) $$PI_{j}=s\left(f,X_{\text{test}},y_{\text{test}}\right)-\frac{1}{K}\sum_{k=1}^{K}s\left(f,X_{\text{test}}^{\left(j,k\right)},y_{\text{test}}\right),$$

which reflects the drop in held-out score when feature *j*’s values are randomly shuffled, i.e., out-of-sample cross-driver relevance rather than in-sample impurity reduction (Equation (9)). Record-wise (ungrouped) cross-validation is known to overstate performance relative to subject-wise (grouped) cross-validation in mobile- and wearable-sensing machine learning generally [79]. In order to quantify the size of this effect for the present data and features specifically, we also ran an ungrouped stratified 5-fold cross-validation. No hyperparameter tuning, feature selection, or normalization fit on the full dataset occurs anywhere in the primary evaluation; the 32 features are fixed hand-crafted quantities computed independently window-by-window before any train/test split, the classifier uses one fixed hyperparameter setting throughout. and each leave-one-driver-out fold’s held-out driver contributes no information, labeled or unlabeled, to that fold’s training step.

*Sensitivity analyses.* Two sensitivity analyses were used to probe the repeated-driver confound. Sensitivity A repeated the leave-one-driver-out random forest evaluation, restricted to the seven drivers who completed more than one route by removing the four Rural-only drivers whose routes were fully confounded with their identity. Sensitivity B used a within-subject paired design: for the five drivers who drove both Highway and Urban (the only pairing with three or more within-subject pairs; the Rural–Highway and Rural–Urban pairings each had only a single driver and so were not statistically testable), we computed each driver’s mean value for every feature on each route and applied a Wilcoxon signed-rank test to the five paired differences, with FDR correction across features.

*Baseline correction ablation (causal, primary).* To test whether the weak leave-one-driver-out accuracy reflects large and stable between-driver baseline differences swamping a smaller signal related to driving context while ensuring a prospective calibration period that avoids leaking each test window’s statistics into its own normalization, we restricted this analysis to the seven multi-route drivers who completed two sessions in a fixed and data-confirmed chronological order (an earlier session, then a later session, always along a different route). For each such driver in turn, the classifier was trained on the other ten drivers’ complete data (each training driver’s features being standardized using that driver’s own full data). A per-feature baseline (mean, standard deviation) was then computed from only that driver’s earlier session; this baseline was used to *z*-score the driver’s later session (Equation (10)), and the classifier prediction used only the later session. This approaches exclusively uses the driver’s own prior unlabeled data for calibration, never the labels or windows being predicted; in this way, it provides a causal 7-fold evaluation, with one fold per multi-route driver.

*Baseline correction ablation (transductive, sensitivity check).* As a sensitivity check, we also report a simpler per-driver standardization used in an earlier version of this analysis, with both results shown side-by-side. Each feature was *z*-scored (within-driver) using that driver’s own mean and standard deviation across all of their own windows, including the windows subsequently used for testing:

(10) $$z_{ijk}=\frac{x_{ijk}-{\bar{x}}_{i}}{s_{i}}$$

where $x_{ijk}$ is the raw value of a feature for driver *i*, route *j*, window *k*, and ${\bar{x}}_{i}$, while $s_{i}$ represents that driver’s own mean and standard deviation for the feature (Equation (10)); features with zero within-driver variance were set to 0 after centering. Because this version’s normalization statistics are computed in part from the same windows later scored as test data, it is transductive rather than causal; it does not leak road_condition labels across leave-one-driver-out folds, but also does not approximate a real prospective calibration period in the way that the causal version does.

*Uncertainty quantification.* Considering that the cross-validation comparison includes only 11 drivers, we quantify the uncertainty by computing 95% bootstrap confidence intervals (3000 resamples, percentile method) for the primary and transductively baseline-corrected leave-one-driver-out accuracy results, the Sensitivity A accuracy, as well as for the naive ungrouped accuracy. For each, we perform a cluster bootstrap to resample drivers with replacement and pool each resample’s out-of-fold predictions before scoring (with windows resampled directly for the naive ungrouped estimate); the causal baseline correction accuracy used the same cluster bootstrap approach, but restricted to the seven multi-route drivers. The gap between the naive and primary accuracy results was bootstrapped by resampling the same set of drivers for both estimates on each iteration and then taking the difference.

### 3.5. Additional Robustness and Diagnostic Analyses

*Feature correlation and multicollinearity.* Permutation importance (Equation (9)) is known to be distorted when input features are highly correlated: importance can be split, suppressed, or arbitrarily assigned among near-duplicate features. Therefore, we computed the pairwise Pearson correlation matrix for all 32 features; for each feature, we also computed its variance inflation factor (VIF) against the remaining features:

(11) $$VIF_{j}=\frac{1}{1-R_{j}^{2}},$$

where $R_{j}^{2}$ is the coefficient of determination from regressing feature *j* on all other standardized features (Equation (11)); VIF >10 is a conventional threshold for problematic multicollinearity. One feature, tags_count, was constant (zero) across every window in the cleaned dataset and was excluded from this analysis, leaving 31 features.

*Effect sizes.* Because $y_{ijk}$ in Equation (5) is standardized to zero mean and unit variance for each feature before the mixed-effects model is fit, the fixed-effect coefficients $\beta_{1}$ and $\beta_{2}$ are themselves approximate standardized mean differences (Cohen’s *d*) for the Highway vs. Rural and Urban vs. Rural contrasts, adjusted for the driver random intercept; refitting Equation (5) with Highway as the reference level recovers the third contrast, Urban vs. Highway, in the same standardized units. We report the largest of the three absolute standardized contrasts per feature, ${\left|d\right|}_{\max}$, alongside its FDR-corrected *p*-value, using the conventional thresholds of |d|≈0.2, 0.5, and 0.8 for small, medium, and large effects. These window-level effect sizes describe the naive model’s contrasts, and are reported for comparability with the existing literature on effect sizes. We do not report an analogous session-level effect size table, since with only 18 sessions and seven model parameters (Equation (7)) the session-level coefficients are themselves too unstable (standardized magnitudes exceeding |d|=3 for several features) to interpret as precise effect sizes; we treat the session-level model’s FDR-corrected omnibus *p*-value (Section 4) as the primary quantity.

*Multi-classifier comparison.* To test whether the leave-one-driver-out accuracy reported in Section 4.4 is specific to the random forest, we repeated the identical leave-one-driver-out evaluation with two additional classifiers under the same feature set, grouping, and scoring (Equation (8)): a logistic regression on standardized features (L2-regularized, balanced class weights) and a gradient-boosted tree ensemble (200 trees, maximum depth 3). No hyperparameter tuning was performed for any of the three classifiers.

*SHAP cross-check.* We computed SHAP (SHapley Additive exPlanations) values for the primary random forest. This approach is increasingly paired with wearable sensor classifiers for feature-level explainability [58], and is intended as a cross-check on permutation importance that is model-agnostic among the different tree models. For a feature *j*, coalition *S* drawn from the full feature set *F*, and model output $f_{x}$, the Shapley value is

(12) $$\phi_{j}\left(x\right)=\sum_{S\subseteq F\setminus \{j\}}\frac{|S|!\;(|F|-|S|-1)!}{|F|!}\left[f_{x}\left(S\cup \left\{j\right\}\right)-f_{x}\left(S\right)\right],$$

i.e., feature *j*’s average marginal contribution to the prediction across all possible orderings of the other features (Equation (12)). SHAP values were computed with a tree-based explainer on each leave-one-driver-out fold’s held-out data using that fold’s own trained model, then averaged in absolute value across folds. We compare the resulting feature ranking to permutation importance (Section 4.7) using Spearman rank correlation and top-10 set overlap.

All analyses were implemented in Python 3.11 using pandas 3.0.2, scikit-learn 1.8.0, statsmodels 0.15.0, SciPy 1.17.1, and SHAP 0.51.0.

## 4. Results

### 4.1. Feature Distributions and Population-Level Statistical Comparison

Twenty-seven of the 32 features differed significantly by driving context at FDR <0.05 in the window-level driver-adjusted mixed-effects models (Equations (5) and (6)). A naive analysis of this dataset would report this result as primary; Section 4.2 reports the corrected session-level result, where we treat only 2 of 32 features as primary. Ranking features by statistical significance alone would be misleading, since a *p*-value conflates the effect magnitude with the statistical power; thus, Table 2 reports standardized effect sizes (Section 3) alongside FDR-corrected *p*-values for the features with the largest and smallest effects. By this standardized measure, the four heart rate variability features (hr_mean_bpm, pnn50_pct, ibi_mean_ms, and peak_count) carry the largest effects (|d|=0.71–0.83, all driven mainly by Rural vs. Highway and Rural vs. Urban contrasts), followed by EDA phasic variability, SCR mean amplitude, and skin temperature slope and mean (|d|=0.52–0.68, driven mainly by the Urban vs. Highway contrast, $p<1\times 10^{-36}$ for the first two). Figure 3 shows distributions for eight representative features spanning all four sensor modalities.

Highway sessions showed the highest mean EDA level and variability (EDA mean 1.47 vs. 1.26 in Urban and 0.26 in Rural; SCR amplitude 0.076 vs. 0.035 and 0.012 respectively), highest heart rate (75.9 vs. 74.6 and 72.2 bpm), and highest skin temperature (33.2 vs. 32.6 and 31.4 °C). SCR count showed the opposite pattern, being highest in Rural (24.9 events per window vs. 17.4 in Urban and 16.9 in Highway). Accelerometer movement count was lowest in Urban (182.9) relative to Rural (213.9) and Highway (215.1), consistent with stop-and-go urban driving producing more sustained low-amplitude motion rather than the count-based movement events captured by this feature.

Two features illustrate why standardized effect sizes are needed to assess practical significance, as opposed to raw coefficients or *p*-values alone. First, temp_slope reached very small *p*-values ($p<1\times 10^{-8}$ for two of three contrasts) with a raw coefficient on the order of $1\times 10^{-14}$ °C per microsecond. This is small enough in unstandardized units that the initial reading of these results judged the effect to be negligible; after standardization, temp_slope’s effect is in fact medium (d=0.60, Table 2), and is the seventh-largest of the 32 features. Judging effect size from the raw coefficient’s units rather than its standardized magnitude made the effect initially appear negligible. This interpretation is further complicated by temp_slope’s near-perfect correlation with temp_mean (r=0.9995, Section 4.3); the two are more properly read as one shared thermoregulatory effect than as two independent findings. Cases of a small effect reaching FDR significance only because of the sample size (1536 windows) include bvp_min, acc_y_std, and temp_std (|d|=0.16–0.19, Table 2), each of which is significant at FDR <0.05 despite a standardized effect below the conventional threshold for even a small effect.

### 4.2. Session-Level Statistical Comparison (Corrected)

Matching the statistical unit to the 18-session experimental unit and correcting the pairwise-multiplicity issue (Section 3) sharply reduces the number of significant features in a way that isolates how much each of the two problems separately contributes to the window-level count of 27. Aggregating to session level while still taking the minimum of three pairwise *p*-values per feature, i.e., fixing only the unit-of-analysis problem, leaves 11 of 32 features significant at FDR <0.05. Replacing the pairwise-minimum procedure with the single cluster-robust omnibus Wald test per feature, i.e., additionally fixing the multiplicity problem, leaves only 2 of 32 features significant: scr_count (omnibus FDR p=0.022) and acc_movement_count (omnibus FDR p=0.036). Both directions match the raw pattern already reported above; session-level scr_count is highest in Rural (mean 24.8 events, SD 6.9) and lowest in Highway (mean 17.0, SD 7.2) and Urban (mean 17.4, SD 6.5), while session-level acc_movement_count is lowest in Urban (mean 183.9, SD 15.0) relative to Highway (mean 216.6, SD 22.0) and Rural (mean 212.9, SD 32.4). Holm-corrected post hoc pairwise contrasts show that scr_count differs between all three driving contexts (all Holm p<0.001), while acc_movement_count’s omnibus significance is driven specifically by the Urban vs. Highway contrast (Holm p=0.0008; Highway vs. Rural and Urban vs. Rural both Holm p=1.0). Because cluster-robust inference with only eleven driver clusters (four being singletons) sits at the edge of reliable small-sample asymptotics, we cross-checked both features with the 5000-draw label-permutation test described in Section 3. Both features held up (scr_count permutation p=0.016; acc_movement_count permutation p=0.012), so the corrected result is not an artifact of small-cluster Wald-test unreliability. We treat this session-level omnibus-tested result as this paper’s primary statistical finding. Together with Table 2, the initial window-level result complements the primary finding by demonstrating the large effect of the uncorrected pseudoreplication and multiplicity issue on this dataset.

### 4.3. Feature Correlation and Multicollinearity

**Figure 4:**
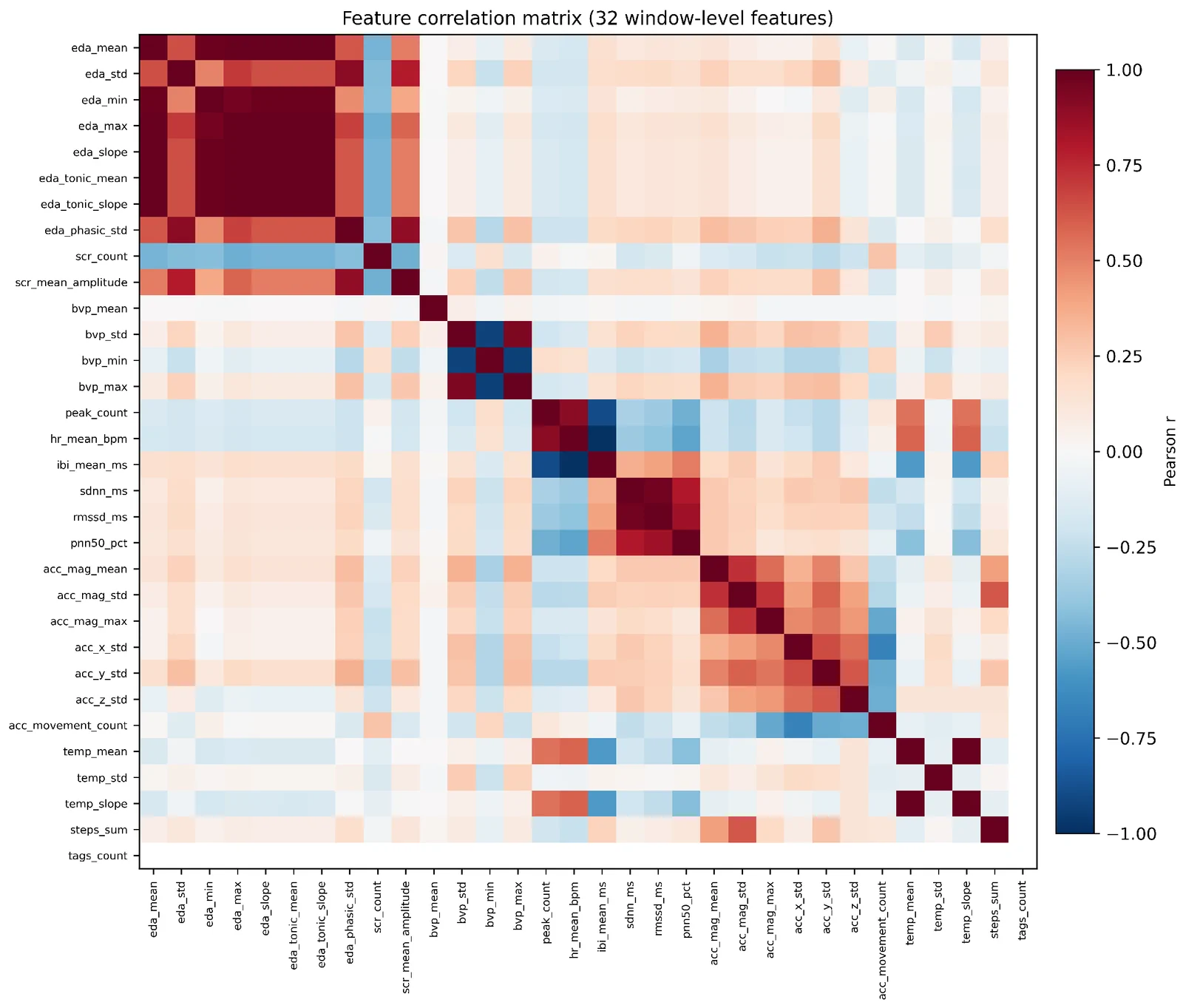
Pairwise Pearson correlation matrix for the 32 window-level features.

Section 3 describes the correlation and variance inflation factor (VIF) diagnostics computed across the 32 features (31 after excluding tags_count, which was constant at zero across every window in the cleaned dataset). Figure 4 shows the full pairwise correlation matrix; 27 feature pairs had |r|>0.8, four of them essentially perfect (r>0.9999), with eda_mean, eda_tonic_mean, eda_slope, and eda_tonic_slope forming a single redundant cluster. This is expected given that the tonic component is a slow-moving transformation of the raw EDA signal and that the mean and slope are computed over the same short window (Equation (1)). Likewise, temp_mean and temp_slope are near-perfectly correlated (r=0.9995), consistent with the temp_slope interpretation in Section 4.

Table 3 reports the VIF for the most collinear features; 17 of the 31 tested features exceed the conventional VIF >10 threshold, including VIF values in excess of $10^{12}$ for the four-feature EDA cluster described above, meaning that this cluster essentially carries one dimension of information rather than four. This has direct implications for interpreting the permutation importance ranking in Section 4.7; with several near-duplicate features present, permutation importance can arbitrarily assign or split credit among them, so ordinal differences within a correlated cluster (for example, ranking eda_mean above eda_tonic_mean) should not be over-interpreted, though the qualitative finding that EDA-family features as a group carry classification-relevant signal is unaffected by which specific member of the cluster is credited.

### 4.4. Classification Performance

**Figure 5:**
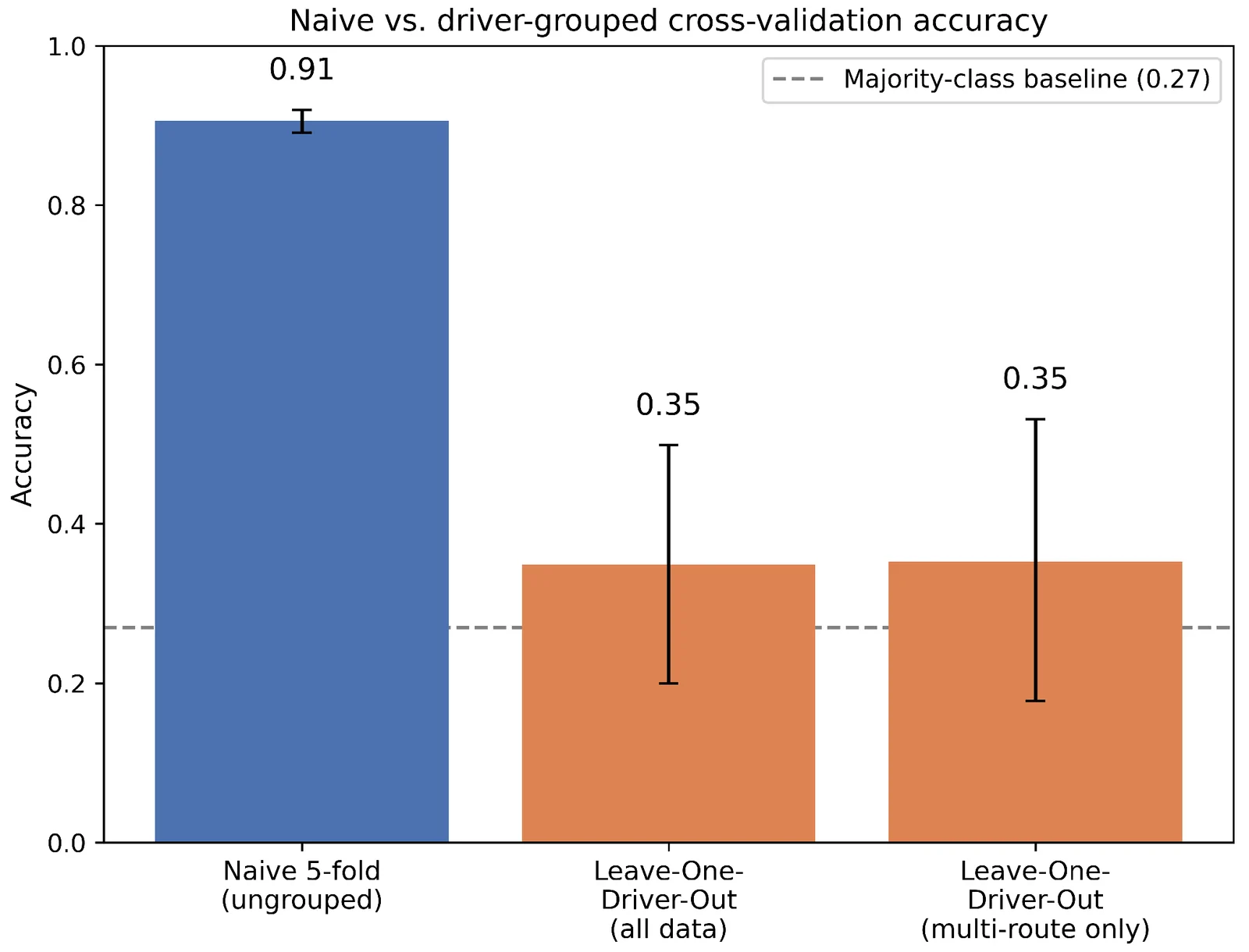
Accuracy under naive (ungrouped) cross-validation versus driver-grouped leave-one-driver-out cross-validation against a majority-class baseline evaluated under the same grouped scheme. Blue: naive ungrouped 5-fold cross-validation; orange: driver-grouped leave-one-driver-out (all 11 drivers, and the seven multi-route drivers only); dashed grey line: majority-class baseline. Error bars: 95% bootstrap confidence intervals (window bootstrap for the naive estimate; cluster bootstrap over drivers for the leave-one-driver-out estimates).

A naive ungrouped 5-fold stratified cross-validation achieved 90.6% accuracy (±2.0%, macro-F1 =0.905; 95% bootstrap CI [89.1%, 92.0%]). The leave-one-driver-out evaluation, which prevents windows from the same driver appearing in both training and test folds, achieved only 34.9% accuracy (macro-F1 =0.33; 95% CI [20.0%, 49.9%]), barely above the majority-class baseline of 27.0% and close to the stratified-random baseline of 32.7% under the identical grouped scheme (Figure 5). The gap between the two is 55.6 percentage points and has a 95% bootstrap CI of [42.0, 69.2] points, making it clearly distinguishable from zero despite the small number of drivers. Accuracy and macro-F1 (Equation (8)) are reported throughout using the standard definitions above.

**Figure 6:**
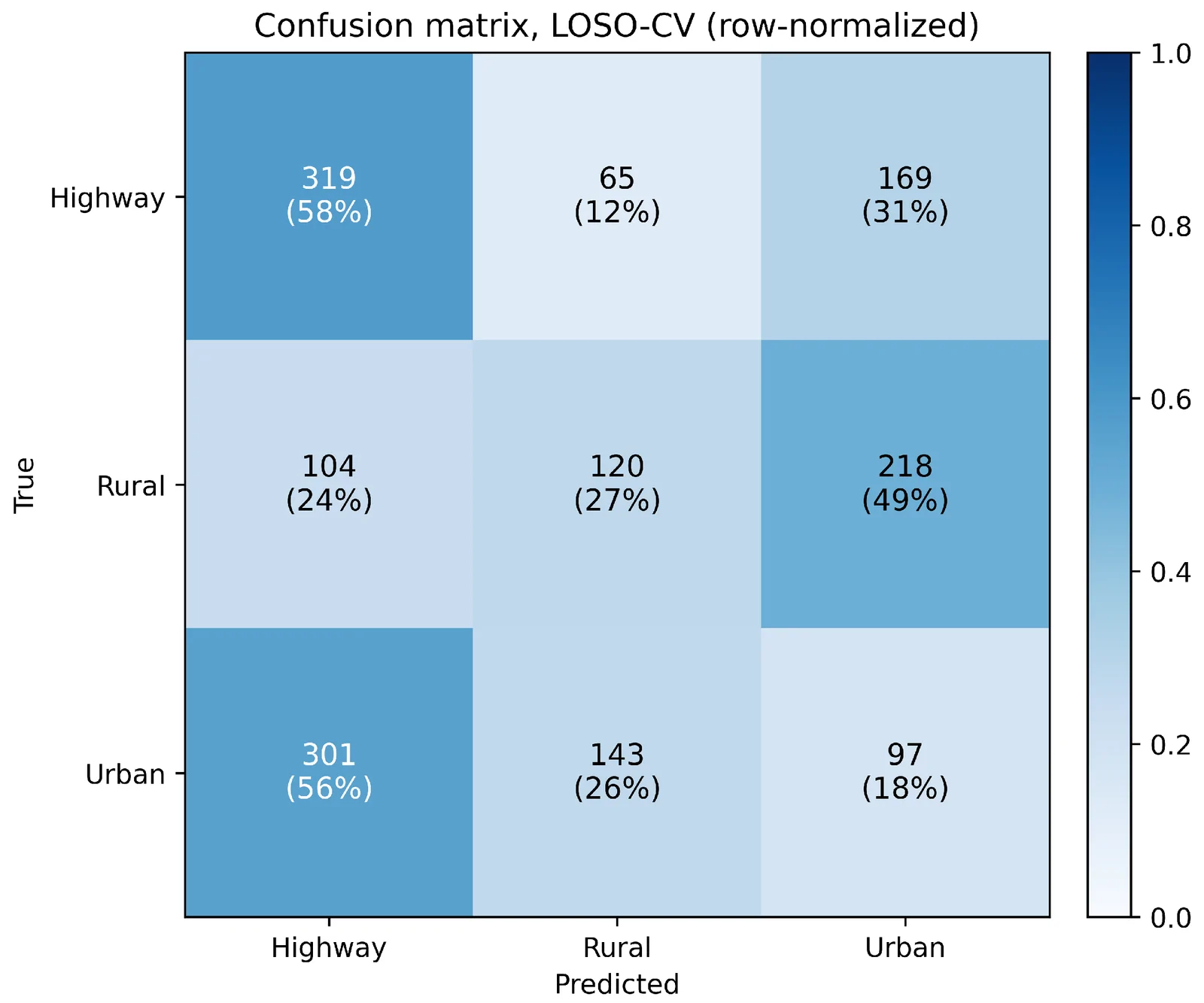
Pooled confusion matrix across the 11 leave-one-driver-out folds (row-normalized percentages in parentheses).

Restricting the leave-one-driver-out evaluation to the seven drivers who completed more than one route (Sensitivity A) gives a similarly modest accuracy of 35.3% (macro-F1 =0.25; 95% CI [17.7%, 53.2%]) against 32.6% for the majority baseline, indicating that this weak generalization is not simply an artifact of the four Rural-only drivers. Per-class performance in the primary model is uneven; Highway had the best recall (57.7% recall, 44.1% precision, F1 =0.50), Rural was intermediate (27.1% recall, 36.6% precision, F1 =0.31), and Urban was the most challenging to identify (17.9% recall, 20.0% precision, F1 =0.19). The balanced accuracy (macro-average of per-class recall) was 34.3%, which is close to the overall accuracy because the three classes are close to equally sized (553, 442, and 541 windows respectively). The pooled confusion matrix (Figure 6) shows the classifier’s predictions concentrated toward Highway regardless of the true class; this was most visible for Urban windows, 301 of which (55.6%) were misclassified as Highway.

### 4.5. Multi-Classifier Robustness Check

**Figure 7:**
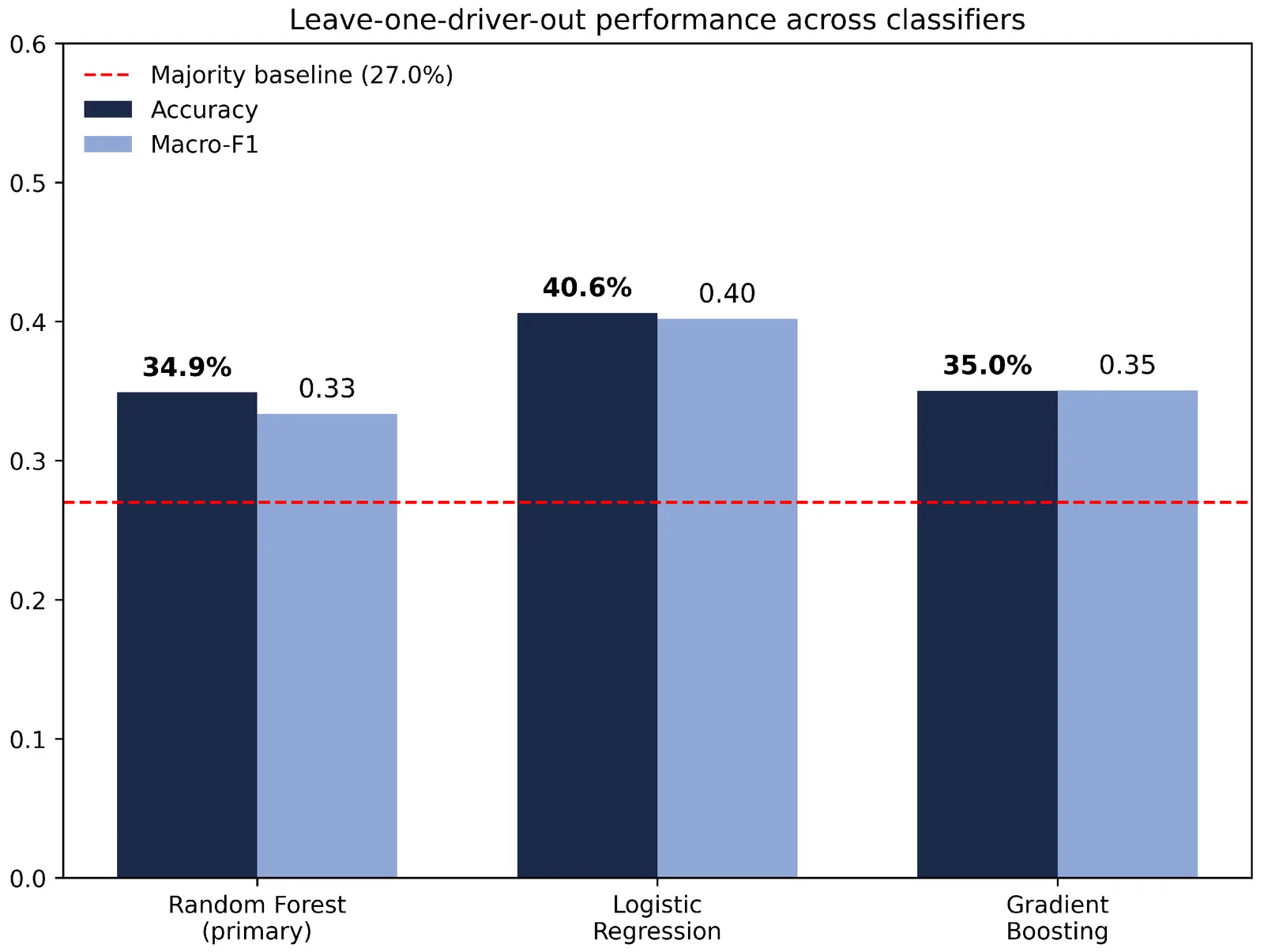
Leave-one-driver-out accuracy and macro-F1 for the random forest, logistic regression, and gradient boosting classifiers against the majority-class baseline.

To test whether the modest leave-one-driver-out accuracy above is specific to the random forest, we repeated the identical evaluation (Section 3) with logistic regression and gradient boosting. All three classifiers were weak relative to the naive ungrouped result, and none approached even half of the naive accuracy (Figure 7, Table 4). Logistic regression reached the highest accuracy of the three at 40.6% (macro-F1 =0.40), gradient boosting closely matched the random forest at 35.0% (macro-F1 =0.35) versus 34.9% (macro-F1 =0.33), and per-fold accuracy was highly variable for all three classifiers (standard deviation 27–31 percentage points across the eleven folds). These results similarly reflect the instability of the small driver sample, rather than being specific to any one algorithm.

The linear model was the simplest classifier we tested, and generalized at least as well as the two tree ensembles. This is consistent with the multicollinearity findings in Section 4.3: tree-based feature selection can behave erratically when choosing among near-duplicate correlated features at each split, while a regularized linear model can combine correlated predictors more smoothly. This is noted as a plausible contributing factor rather than a confirmed mechanism; isolating it would require a dedicated ablation. The more central conclusion of this comparison is that the weak leave-one-driver-out accuracy reported in Section 4.4 is not an artifact of the random forest model specifically.

### 4.6. Modality Ablation

Table 5 reports the leave-one-driver-out accuracy for each of the four feature subsets described in Section 3. No single modality dominates; accelerometry alone (38.1%, macro-F1 =0.38) outperformed physiology alone (32.8%, macro-F1 =0.31), temperature alone (35.9%, macro-F1 =0.35), and even the full 31-feature combined model (33.9%, macro-F1 =0.33), which did not outperform accelerometry alone despite having access to all of its features plus 23 more. The per-fold accuracy for accelerometry alone was also the most stable of the four (SD 11.5 percentage points versus 20–32 points for the other three), while physiology and the combined model showed the same high fold-to-fold variability seen throughout the classification results above.

This result speaks directly to the collinearity concern raised in Section 4.3: if driving context leaves a physiology-specific signature that accelerometry and temperature only partially share, the combined model should outperform every single-modality model, since it has more information available; however, it does not. Thus, a more consistent account is that a substantial share of what each modality captures is a shared cross-modal correlate of driving context, plausibly including route duration, average speed, or stop-and-go pattern (Section 3, Dataset), rather than three independent modality-specific channels of road-related information. Because combining the channels adds redundant rather than complementary signal, it can slightly dilute the strongest single channel (accelerometry) with noisier ones. In Section 5, we return to this point and address its implications for interpreting the population-level statistical results.

### 4.7. Feature Importance

**Figure 8:**
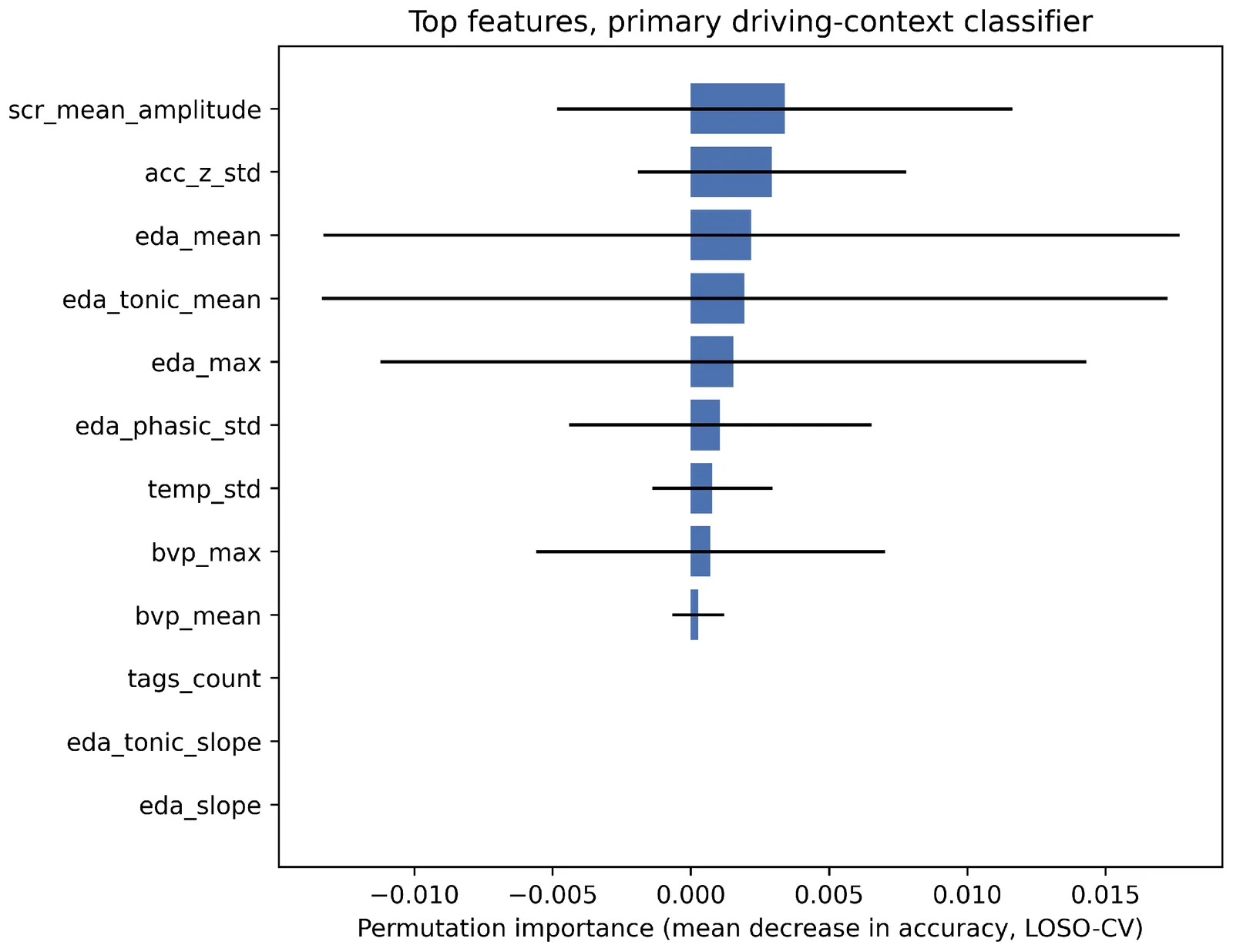
Top features by permutation importance (Equation (9)) averaged across the 11 leave-one-driver-out folds (error bars: standard deviation across folds).

Under the leave-one-driver-out scheme, permutation importance (Equation (9); Figure 8) identified the SCR mean amplitude and z-axis accelerometer standard deviation as the two most consistently useful features for out-of-sample prediction, followed by several EDA-level features (mean, tonic mean, max, phasic standard deviation), temperature variability, and BVP extremes. As shown in Section 4.3, several of these EDA features are near-perfectly collinear, so their individual rank order should be read as identifying an informative feature cluster rather than single cluster member. Despite HRV features (heart rate, IBI, SDNN, RMSSD, pNN50) being among the most statistically significant and the largest by standardized effect size, the features in the population-level mixed-effects analysis (Section 4) contributed little to cross-driver classification importance. This suggests their driving context association is confounded with or dominated by stable between-driver differences that the leave-one-driver-out design correctly discounts.

**Figure 9:**
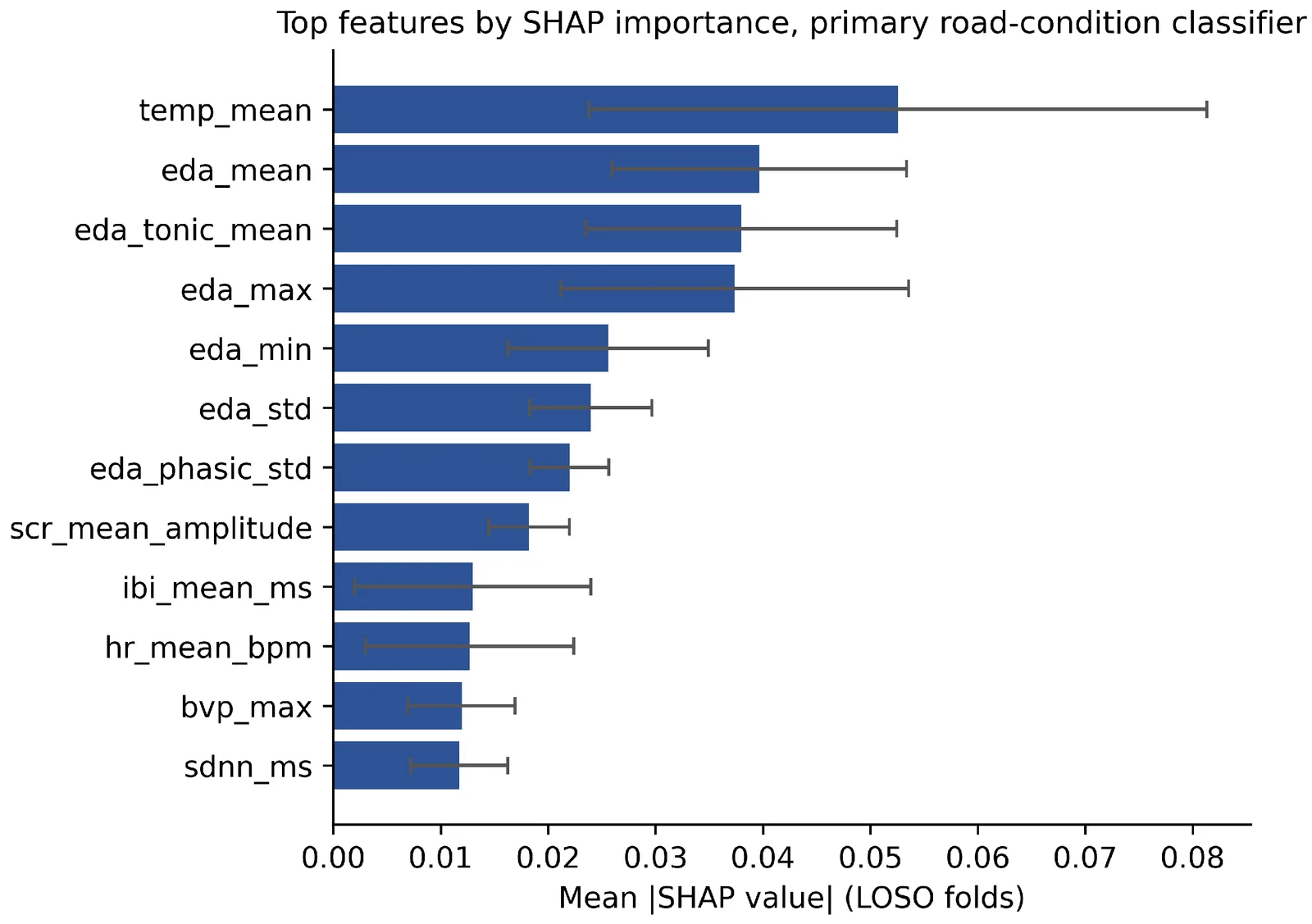
Top features by mean absolute SHAP value (Equation (12)) averaged across the 11 leave-one-driver-out folds (error bars: standard deviation across folds).

As a cross-check on this ranking, we computed SHAP values for the primary random forest on each leave-one-driver-out fold’s held-out data (Equation (12), Section 3; Figure 9). The two importance measures showed only partial agreement: five of the ten highest-ranked features by permutation importance also appeared in the top-10 SHAP ranking, and both rankings were dominated by EDA-family features, consistent with Section 4.3. However, the overall rank correlation between the two measures across all 32 features was effectively zero (Spearman ρ=−0.0004, p=0.998). SHAP ranked temp_mean and the four collinear EDA features (eda_mean, eda_tonic_mean, eda_max, eda_min) above scr_mean_amplitude, which permutation importance had ranked first. SHAP also assigned negligible importance to tags_count, the consistently uninformative feature identified in Section 4.3, whereas permutation importance ranked it in the top 10 (Figure 8) simply because several other features had comparably close-to-zero importance in that scheme. SHAP’s ranking separates a truly uninformative feature from borderline ones in a way that the permutation importance ranking does not make visually apparent. Taken together, the two importance measures agree on the qualitative picture, where EDA family and temperature features dominate the cross-driver transferable signal and HRV contributes comparatively little; however, they disagree enough in terms of the detailed rank order that we treat neither ranking’s exact ordering as more than illustrative, particularly within the collinear EDA cluster.

### 4.8. Within-Subject Sensitivity Analysis

Among the five drivers who completed both the Highway and Urban routes, seven features (EDA standard deviation, EDA phasic standard deviation, SCR mean amplitude, accelerometer magnitude maximum, z-axis and x-axis accelerometer standard deviation, and movement count) showed the same direction of difference for all five drivers (Wilcoxon W=0, nominal p=0.0625, the smallest attainable *p*-value with five pairs). None survived FDR correction across the 32 features tested (all corrected p>0.28), reflecting the limited statistical power of a five-pair design rather than an absence of effect. The Rural vs. Highway and Rural vs. Urban comparisons each rest on a single driver (SUB1 and SUB6, respectively), and so were treated as descriptive-only.

### 4.9. Baseline Correction Ablation

*Causal prior-session-only calibration (primary).* Restricting the analysis to the seven multi-route drivers and calibrating each driver’s later session using only their own earlier session (Section 3) gave 45.2% accuracy (macro-F1 =0.21) across 613 test windows, with a 95% cluster bootstrap CI of [19.3%, 70.6%]. This evaluation is not directly comparable to the primary leave-one-driver-out results with eleven drivers and three classes because for six of the seven drivers multi-route drivers the later session happened to be an Urban session (the seventh driver’s later session was Highway); thus, the resulting test set contains no Rural windows at all, which both narrows the classification problem and accounts for the low macro-F1 despite a numerically higher accuracy than the primary model. Per-driver accuracy on this 7-fold evaluation ranged from 1.4% to 97.9%, reflecting how much a single held-out driver can swing a 7-fold estimate. Therefore, we report this result here as a directionally consistent but small-sample and structurally class-imbalanced causal calibration check.

**Figure 10:**
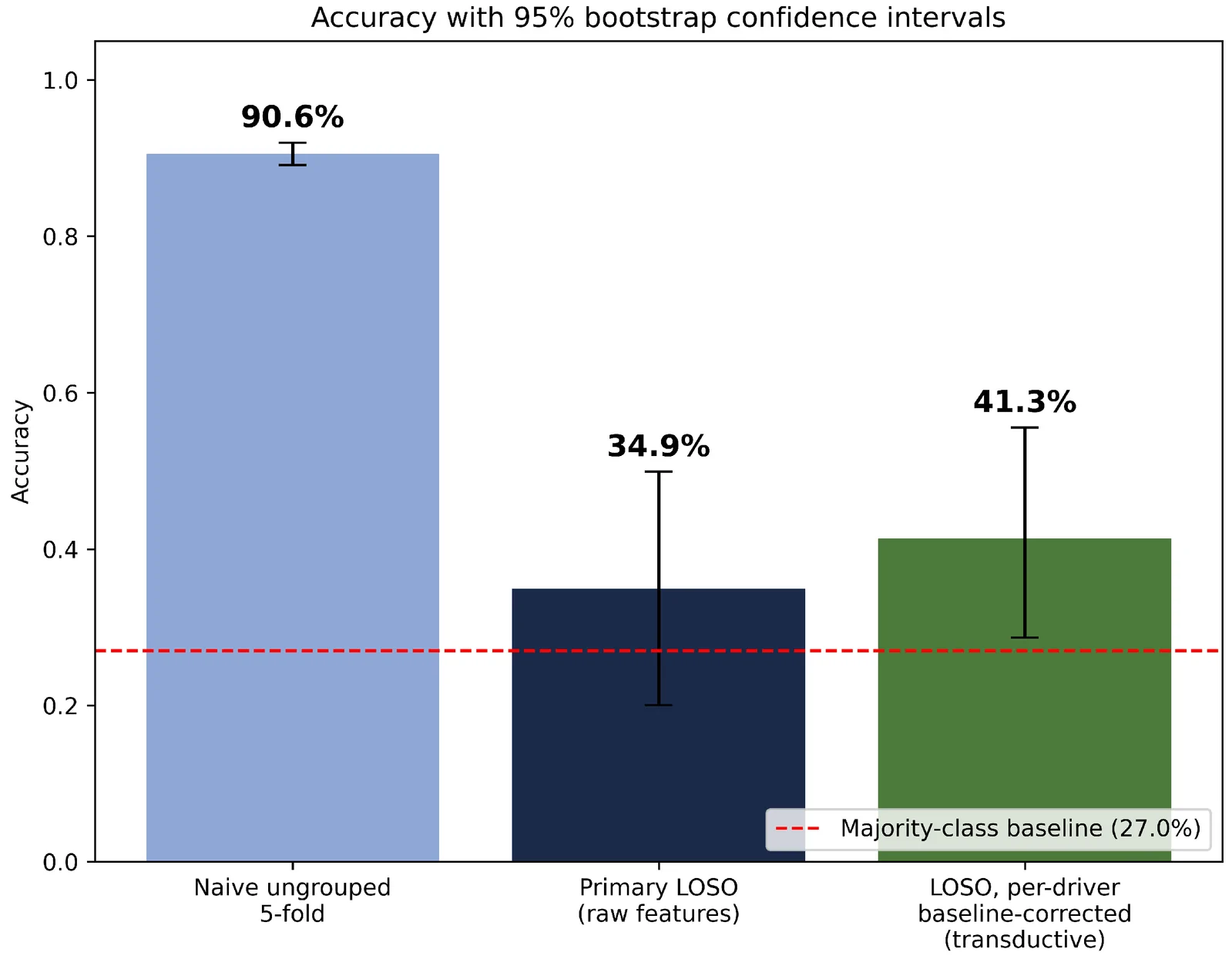
Accuracy for the naive ungrouped, primary leave-one-driver-out, and transductive per-driver baseline-corrected leave-one-driver-out evaluations, with 95% bootstrap confidence intervals and the majority-class baseline.

*Transductive per-driver standardization (sensitivity check).* The simpler non-causal per-driver *z*-scoring described in Section 3 (Equation (10)), which normalizes each driver’s windows using statistics computed over all of that driver’s data, including the windows later used for testing, raised leave-one-driver-out accuracy from 34.9% to 41.7% (macro-F1 from 0.33 to 0.41) across all eleven drivers and all three classes, a 6.8-percentage-point improvement with a 95% bootstrap CI of [28.7%, 55.5%] for the corrected estimate (Figure 10). This is directionally consistent with the hypothesis that stable between-driver baseline differences suppress cross-driver generalization, matching the causal result’s direction above. However, because its normalization statistics are not restricted in time to data available before the test window, it should be read only as a sensitivity check on that hypothesis, not as an estimate of what a real prospective calibration period would achieve; the causal result is the analysis intended for that purpose. The confidence intervals for the raw and transductively corrected leave-one-driver-out accuracy results overlap substantially, so even this simpler comparison should be read as suggestive rather than statistically confirmed at this sample size.

## 5. Discussion

This study asked whether driving context can be classified or meaningfully distinguished from wearable physiological and motion signals alone. The answer is qualified, and depends on which statistical model is treated as primary. At the window level, treating the 1536 windows as independent observations, the majority of features tested (27 of 32) differ significantly across Rural, Highway, and Urban driving; but this is the naive analysis, and once the statistical unit is matched to the 18-session experimental unit and pairwise multiplicity is properly controlled (Section 4.2), only 2 of 32 features (skin conductance response (SCR) count and accelerometer movement count) survive. This corrected session-level result is the paper’s primary statistical finding: driving context leaves a real signature in wrist-worn physiological and motion signals, but this is much narrower than the broad 27-feature signature suggested by the naive window-level analysis. This finding is broadly consistent with the one directly comparable prior result in this space [59], where a single-subject 21-day longitudinal study found that EDA from an Empatica E4 could classify route type at roughly 82.9% accuracy. Because that design used one driver, its high accuracy reflects within-subject decodability rather than cross-driver population-level generalizability, so the two results are compatible rather than contradictory: route type is strongly decodable from EDA within an individual, while a signature that survives correct statistical treatment across individuals is considerably narrower. A classifier trained to generalize to an unseen driver achieves only modest accuracy (34.9%, little better than chance for this three-class problem), which holds even when restricting the analysis to those drivers who contributed data from more than one route. Taken together, our results suggest that much of the driving context-related variance visible at the naive window level is carried on top of large and systematic between-driver differences in baseline physiology and driving style. The leave-one-driver-out classifier cannot exploit this for a new individual, and a substantial share of the naive window-level statistical signal reflects the same pseudo-replication problem rather than 27 independent real effects. The baseline correction ablation (Section 4) tests the between-driver baseline account, lending some support in both its causal and transductive versions: a causal prior-session-only calibration restricted to the seven multi-route drivers raised accuracy to 45.2% on a smaller class-imbalanced 7-fold evaluation, while a simpler non-causal (transductive) per-driver standardization raised the full 11-driver accuracy to 41.7%. Neither improvement is fully distinguishable from the uncorrected estimate at this sample size, so both are best read as suggestive rather than confirmatory, and the causal version specifically should be read alongside its very small class-imbalanced test set rather than as a like-for-like improvement on the primary 34.9% figure.

The gap between the naive ungrouped cross-validation result (90.6% accuracy) and the driver-grouped result (34.9%) is another central finding of this study. The parallel gap in the statistical results (27 of 32 features nominally significant at the window level, but only 2 of 32 after the statistical unit and pairwise multiplicity are corrected) is the same phenomenon, just appearing in a different analysis. A 5-fold ungrouped split allows windows from the same driver and even the same session to appear in both the training and test partitions; because consecutive 60-s windows from one drive are highly autocorrelated and carry a stable individual physiological fingerprint, a classifier evaluated in this way can achieve high accuracy largely by recognizing the driver (and, by extension, which route that driver happened to be recorded on) rather than by learning a context-general signature. Both patterns are specific instances of a broader and already well-established pseudo-replication risk in the mobile and wearable sensing machine learning literature. Record-wise cross-validation is known to overstate real-world performance relative to subject-wise cross-validation [79], and our results extend this caution from classification accuracy to statistical significance testing in the setting of classifying driving context from wearables. The literature on wearable-based driver monitoring does not yet treat grouped evaluation or session-matched statistical testing as universal practice, so reported accuracy and *p*-value results in this literature should both be read alongside their unit of analysis. Designs that do not explicitly group by participant, or that test at the window level while applying the experimental manipulation at the session level, are prone to substantial overstating of both real-world cross-driver performance and statistical significance.

The features that proved most useful for cross-driver generalization were SCR amplitude and z-axis accelerometer variance (Section 4.7), and the two features that survive the corrected session-level statistical test were SCR count and accelerometer movement count (Section 4.2). Although the specific measures differ, all of these are drawn from the same two feature families, namely, electrodermal and accelerometer. Both analyses agree that despite HRV dominating the naive window-level significance ranking (Table 2), it contributes comparatively little when the statistical unit is corrected or when cross-driver classification is the criterion rather than within-sample fit. A plausible explanation for these results is that HRV features in particular reflect a driver’s resting autonomic baseline; as they change relatively slowly, HRV values for a 60-s window provide more information about who is driving than about the driving context. The window-level mixed-effects model can still detect a population-level association between HRV and driving context if the sample of drivers on each route differs systematically (as it does here, since not all drivers drove all routes), which is exactly the kind of spurious unit-of-analysis-driven association that the session-level correction is designed to remove. By contrast, SCR-family features and accelerometer movement/variance are more proximally tied to momentary events such as startle response to a hazard, bumps, hard turns, or lane changes. Such events may recur across drivers on the same route type even though their overall arousal or motion baselines differ, which is consistent with their surviving both the cross-driver classification test and the session-level statistical test while HRV survives neither.

The modality ablation (Section 4.6) adds a further piece to this picture. If driving context left a physiology-specific signature that EDA and HRV carried and that accelerometry and temperature only partially shared, then the combined-modality classifier should outperform every single-modality classifier, since it strictly dominates each in available information. However, it does not; accelerometry alone (38.1%) outperforms the 31-feature combined model (33.9%), and no modality is clearly dominant. In the session-level statistical result, the two surviving features come from the accelerometer and electrodermal families specifically. In the mediating-pathway framing introduced above, physiological sensors capture a driver’s response to context as opposed to the context itself; in turn, such responses are plausibly mediated by workload, arousal, or situational awareness demands that differ systematically across a monotonous highway, a rural loop, and a stop-and-go urban corridor [17,18]. In this way, the driving context produces a diffuse cross-modal physiological and behavioral response, rather than a physiology-specific signature that the wrist-worn EDA or HRV sensor captures. This is consistent with the broader domain generalization literature on physiological driver state estimation, where generalizing across drivers is recognized as a harder and more practically relevant problem than achieving high within-driver accuracy [67].

When compared with the established literature on road surface and terrain classification from accelerometer signals (Section 2), which reports strong performance from vehicle- or platform-mounted inertial sensors, the modest performance found here for a wrist-worn wearable is consistent with the sensor being decoupled from the vehicle chassis by the arm and torso, as well as with wrist motion reflecting voluntary gestures and steering wheel interaction along with vehicle dynamics. As noted in the Introduction, the literature on road surface condition also targets a different and more objective construct than the route and driving context categories used here, so the comparison is illustrative rather than a like-for-like benchmark. It is also consistent with the literature on wearables for drowsiness detection, which similarly finds EDA and HR features to be informative but noisy and strongly dependent on the individual. Our results extend the same caution to the driving context inference problem studied here.

Two additional analyses temper how these feature-level conclusions should be read. First, several of the features behind both the window-level statistical comparison and the classifier’s importance ranking are severely collinear (Section 4.3), most notably a four-feature EDA cluster with variance inflation factors exceeding $10^{12}$. This does not undermine the finding that the EDA-family signal differs by driving context, but does mean that crediting one specific EDA feature over another in either the window-level ranking (Table 2) or the permutation importance ranking (Section 4.7) is not well-supported by this design and should be read at the level of the feature family rather than the individual measurement. The session-level correction (Section 4.2) sidesteps this specific issue by testing a single hypothesis per feature rather than ranking correlated features against each other. Second, the SHAP cross-check (Section 4.7) agrees with permutation importance on the broad picture; EDA and temperature features dominate, while HRV contributes little to cross-driver generalization. However, there is essentially no rank correlation in the fine-grained ordering (Spearman ρ≈0). Combined with the multi-classifier comparison (Section 4), where a simple logistic regression matched or slightly exceeded both tree ensembles, and the modality ablation in which no single sensor channel or their combination clearly dominated, this is consistent with a data generation process in which several correlated feature families carry overlapping information about a diffuse cross-modal driving context signal; different methods that are individually reasonable can distribute credit among them differently without any one ranking being more correct than another. Therefore, we treat the session-level statistical result and the classification and modality ablation results as this study’s robust primary conclusions, and treat the individual-feature rankings in Table 2, Figure 8 and Figure 9 as illustrative.

## 6. Limitations

Several limitations qualify these findings. The sample is small (11 drivers, 18 sessions), and four of the 11 drivers drove only the Rural route, so driving context and individual identity are perfectly confounded for those drivers in the full-data model. The Sensitivity A analysis, which was restricted to the seven multi-route drivers, addresses this only partially, since even those drivers each contributed a fixed and non-overlapping set of routes. The one genuinely unconfounded design available in this dataset is the within-subject paired comparison, which is limited to five driver pairs for the Highway vs. Urban contrast and only one pair each for the two Rural contrasts, giving the study limited power to confirm within-person effects that the window-level and cross-driver analyses suggest. The small number of drivers is directly visible in the width of the bootstrap confidence intervals reported in Section 4 (e.g., [20.0%, 49.9%] for the primary leave-one-driver-out accuracy) and in the session-level model’s reliance on only 11 driver clusters (four of them singleton) for cluster-robust inference. Even after the permutation cross-check in Section 4.2, this sits at the edge of reliable small-sample asymptotics; a larger driver sample, specifically with more multi-route drivers, would be needed in order to more precisely estimate cross-driver accuracy and session-level significance and confirm whether either version of the baseline correction ablation’s improvement is real as opposed to sampling variation. Windows are non-overlapping within a session but remain temporally autocorrelated; moreover, grouping by driver does not fully remove session-level leakage when a driver contributes only one session per route, since a held-out driver’s route was still recorded in a single continuous drive. The sample is also demographically unbalanced (9 of 11 drivers male, 9 of 11 in the 18–35 age group), limiting generalizability to other driver populations. The participants also self-selected which wrist to use for the wearable (predominantly the left wrist; Section 3); given documented left–right lateralization in wrist-worn electrodermal activity [34], this represents an additional uncontrolled source of between-driver variability specific to the EDA feature family.

With over 1500 windows, the window-level mixed-effects models (Section 4) can flag statistically significant differences at very small effect sizes. As Section 4.2 shows directly, it can do so at effect sizes that do not survive correction for the true 18-session unit of analysis. Table 2 shows the small effect size version of this for bvp_min, acc_y_std, and temp_std, each of which is FDR-significant at the window level despite a standardized effect below the conventional threshold for even a small effect. Window-level *p*-values in this paper should always be read alongside both the corresponding effect size and the session-level result, not in isolation. The severe multicollinearity documented in Section 4.3, together with the divergence between permutation importance and SHAP in Section 4.7, is a related caution specific to the feature importance results. The rankings of individual features in this study should be treated as identifying informative feature families (electrodermal activity, temperature, accelerometry, and cardiovascular) rather than as precise and individually reliable orderings. The multi-classifier comparison and modality ablation in Section 4 confirm that the classification result itself (though not necessarily every downstream feature ranking) is not an artifact specific to the random forest or to any one sensor channel. The signal quality criterion applied in Section 3 is a simple proxy for the minimum valid beat count on peak_count, not a full waveform-quality index; it removed a small and isolated set of clearly anomalous windows, but would not catch subtler forms of motion artifact that leave peak_count intact.

Both versions of the per-driver baseline correction in Section 4 also assume that a calibration period is available for a new driver (real and prospective in the causal version, all of the driver’s own data in the transductive version); the causal version’s small class-imbalanced 7-fold evaluation means that its 45.2% figure should be treated as a preliminary and directionally suggestive estimate rather than a validated cold-start-free calibration procedure. We used hand-crafted summary features with a random forest rather than end-to-end deep learning on raw or lightly processed signals. Recent work on sensing via wearables has begun moving toward learned representations, including large pretrained (foundation) models for photoplethysmography and related physiological signals, and it remains an open question whether such representations would narrow the cross-driver generalization gap reported here, though our sample is likely too small to train or fine-tune one from scratch. Finally, weather and traffic conditions were not independently measured session-by-session, only the route-level Google Maps figures reported in Section 3 (Dataset); thus, some of the driving context differences reported here may be partially confounded with these unmeasured session-specific factors beyond what trip duration, route order, sleep, and pre-drive sleepiness (Section 3) can adjust for. No hyperparameter tuning was performed for any of the three classifiers compared in Section 4, although the size of the gap between naive and grouped cross-validation is unlikely to be substantially closed by tuning alone.

## 7. Conclusions

Wearable EDA, cardiovascular, motion, and thermoregulatory signals from an Empatica EmbracePlus differ by driving context at the window level (27 of 32 features, FDR <0.05), but this naive analysis substantially overstates the population-level signal. Once the statistical unit is matched to the 18-session experimental unit and pairwise multiplicity is properly controlled using a single omnibus test per feature, only 2 of 32 features (skin conductance response count and accelerometer movement count) remain significant. This corrected result is the paper’s primary statistical finding. A driver-independent classifier trained on this 11-driver dataset achieves modest accuracy (34.9%, 95% CI [20.0%, 49.9%]), only slightly above a majority-class baseline. This is corroborated by two additional classifiers evaluated under the identical scheme (Section 4), indicating that it is not an artifact of the random forest specifically. Naive ungrouped cross-validation on the same data and features would suggest nearly three times higher performance (90.6%). A bootstrap confidence interval confirms that this gap is not attributable to chance, mirroring the gap between window level and session level in the statistical results above. A four-way modality ablation found that none of the physiological, accelerometric, or thermoregulatory sensor channels dominated and that the combined-channel model did not outperform accelerometry alone. This result is consistent with a diffuse cross-modal driving context signature, plausibly mediated by workload, arousal, or situational awareness demands [17,18], rather than with a physiology-specific one. A causal prior-session-only baseline correction analysis restricted to the seven multi-route drivers raised accuracy to 45.2% on a small class-imbalanced 7-fold evaluation, while a simpler non-causal (transductive) version raised the full 11-driver accuracy to 41.7%. Both of these results are directionally consistent with a between-driver baseline account of the cross-driver generalization gap, but neither is confirmatory at this sample size. A multicollinearity diagnostic (Section 4.3) and a SHAP-based cross-check on feature importance (Section 4.7) further show that the individual feature rankings behind these results are less stable than the session-level, classification-level, and modality-level conclusions, and so should be read at the level of feature families rather than individual measurements. At present, driving context inference from a wrist-worn wearable appears useful as a narrow and correctly unit-matched population-level statistical finding covering EDA-family arousal and coarse movement counting. Whether the modest cross-driver signal would be strengthened with a larger and more balanced sample of drivers and whether individual-specific calibration of the kind explored here in a preliminary way can recover useful classification performance are open questions for future work with more multi-route drivers per participant. Reported findings from naive, window-level, or ungrouped-cross-validation designs in this broader literature should be read in light of the size of these corrections, i.e., roughly threefold for classification accuracy and more than tenfold for the number of nominally significant features.

## Figures and Tables

**Table 1:** Clean (post-exclusion) 60-s window counts by driver and driving context.

Driver	Rural	Highway	Urban	Multi-Route?
SUB1	91	72	0	Yes
SUB2	67	0	0	No
SUB3	63	0	0	No
SUB4	64	0	0	No
SUB5	69	0	0	No
SUB6	88	0	87	Yes
SUB7	0	138	91	Yes
SUB8	0	113	94	Yes
SUB9	0	77	81	Yes
SUB10	0	69	104	Yes
SUB11	0	84	84	Yes

**Table 2:** Standardized effect sizes (${\left|d\right|}_{\max}$, the largest of the three pairwise contrasts, Section 3) for the 15 features with the largest effects and the three features with the smallest FDR-significant effects, alongside FDR-corrected *p*-values. Effect labels use conventional thresholds (|d|≈0.2/0.5/0.8 for small/medium/large; below 0.2 labeled negligible). Full results for all 32 features are included with the released analysis code (see the Data Availability Statement).

Feature	FDR *p*	${\left|d\right|}_{\max}$	Effect
hr_mean_bpm	$1.7\times 10^{-25}$	0.83	Large
pnn50_pct	$1.3\times 10^{-20}$	0.82	Large
ibi_mean_ms	$2.9\times 10^{-25}$	0.80	Large
peak_count	$1.8\times 10^{-16}$	0.71	Medium
eda_phasic_std	$1.3\times 10^{-57}$	0.68	Medium
scr_mean_amplitude	$4.8\times 10^{-42}$	0.65	Medium
temp_slope	$3.3\times 10^{-20}$	0.60	Medium
eda_std	$3.4\times 10^{-36}$	0.54	Medium
temp_mean	$8.6\times 10^{-16}$	0.52	Medium
rmssd_ms	$2.7\times 10^{-7}$	0.46	Small
sdnn_ms	$1.5\times 10^{-5}$	0.39	Small
acc_movement_count	$6.9\times 10^{-9}$	0.36	Small
acc_mag_std	$8.2\times 10^{-4}$	0.35	Small
acc_x_std	$9.8\times 10^{-8}$	0.33	Small
eda_max	$9.3\times 10^{-14}$	0.26	Small
bvp_min	$9.0\times 10^{-4}$	0.19	Negligible
temp_std	$3.0\times 10^{-2}$	0.17	Negligible
acc_y_std	$8.9\times 10^{-3}$	0.16	Negligible

**Table 3:** Variance inflation factors (VIF, Equation (11)) for the most collinear features; 17 of 31 tested features exceed the conventional VIF >10 threshold for problematic multicollinearity.

Feature	VIF	Correlated with
eda_mean	$9.7\times 10^{12}$	eda_tonic_mean, eda_slope, eda_tonic_slope
eda_tonic_mean	$9.7\times 10^{12}$	eda_mean, eda_slope, eda_tonic_slope
eda_slope	$9.7\times 10^{12}$	eda_mean, eda_tonic_mean, eda_tonic_slope
eda_tonic_slope	$9.7\times 10^{12}$	eda_mean, eda_tonic_mean, eda_slope
temp_slope	1462	temp_mean
temp_mean	1456	temp_slope
eda_max	447	–
eda_min	142	–
hr_mean_bpm	55.7	–
ibi_mean_ms	49.3	–
rmssd_ms	22.0	–
sdnn_ms	18.5	–
eda_phasic_std	15.9	–
eda_std	14.6	–
bvp_max	11.8	–

**Table 4:** Leave-one-driver-out performance across three classifier families under the identical evaluation scheme (Section 3).

Classifier	Accuracy	Macro-F1	Per-Fold Accuracy (Mean ± SD)
Random forest (primary)	34.9%	0.33	37.5% ± 31.3%
Logistic regression	40.6%	0.40	44.8% ± 27.0%
Gradient boosting	35.0%	0.35	39.4% ± 30.0%
Majority-class baseline	27.0%	–	–

**Table 5:** Leave-one-driver-out performance by sensor modality (Section 3) against the majority-class baseline (27.0%).

Modality	# Features	Accuracy	Macro-F1	Per-Fold Accuracy (Mean ± SD)
Physiology (EDA + BVP/HRV)	20	32.8%	0.31	36.0% ± 31.9%
Accelerometry	8	38.1%	0.38	37.2% ± 11.5%
Temperature	3	35.9%	0.35	36.9% ± 20.9%
All modalities combined	31	33.9%	0.33	36.9% ± 31.2%

## Data Availability

The raw EmbracePlus sensor recordings underlying this study contain timing and session information that could be identifying for the participating drivers, and are not publicly available; restricted access is imposed for participant privacy and consent reasons. The de-identified window-level feature dataset analyzed in this study (1536 windows by 32 features, with route label and de-identified driver ID) and the analysis code used to produce every statistic, table, and figure reported in this paper are available from the corresponding author upon reasonable request, and will be deposited in a public repository upon publication.
